# Human microbiota-transplanted C57BL/6 mice and offspring display reduced establishment of key bacteria and reduced immune stimulation compared to mouse microbiota-transplantation

**DOI:** 10.1038/s41598-020-64703-z

**Published:** 2020-05-08

**Authors:** Randi Lundberg, Martin F. Toft, Stine B. Metzdorff, Camilla H. F. Hansen, Tine R. Licht, Martin I. Bahl, Axel K. Hansen

**Affiliations:** 1 Department of Veterinary and Animal Sciences, Faculty of Health and Medical Sciences, University of Copenhagen, 1871 Frederiksberg C, Denmark; 2 Internal Research and Development, Taconic Biosciences, 4623 Lille Skensved, Denmark; 3 Present Address: Chr. Hansen, 2970 Hoersholm, Denmark; 4 Present Address: QM Diagnostics, 6534 AT Nijmegen, The Netherlands; 5 National Food Institute, Technical University of Denmark, 2800 Kgs. Lyngby, Denmark

**Keywords:** Microbiome, Immunological disorders, Inflammatory diseases

## Abstract

Transplantation of germ-free (GF) mice with microbiota from mice or humans stimulates the intestinal immune system in disparate ways. We transplanted a human microbiota into GF C57BL/6 mice and a murine C57BL/6 microbiota into GF C57BL/6 mice and Swiss-Webster (SW) mice. Mice were bred to produce an offspring generation. 56% of the Operational Taxonomic Units (OTUs) present in the human donor microbiota established in the recipient mice, whereas 81% of the C57BL/6 OTUs established in the recipient C57BL/6 and SW mice. Anti-inflammatory bacteria such as *Faecalibacterium* and *Bifidobacterium* from humans were not transferred to mice. Expression of immune-related intestinal genes was lower in human microbiota-mice and not different between parent and offspring generation. Expression of intestinal barrier-related genes was slightly higher in human microbiota-mice. Cytokines and chemokines measured in plasma were differentially present in human and mouse microbiota-mice. Minor differences in microbiota and gene expression were found between transplanted mice of different genetics. It is concluded that important immune-regulating bacteria are lost when transplanting microbiota from humans to C57BL/6 mice, and that the established human microbiota is a weak stimulator of the murine immune system. The results are important for study design considerations in microbiota transplantation studies involving immunological parameters.

## Introduction

The gut microbiota is an important component of human health. For studying its role in health and disease, aiming for the development of microbiota-targeting therapeutics, food products and ingredients, mice transplanted with human microbiotas (HMs) have been described and applied for several decades^1–6^, although concerns pertaining to limitations of this model system have been raised^7–10^. Often such models are only studied on their phenotypic expression and the microbiota is not characterized, or the opposite is the case^8^.

Laboratory rodents are routinely raised in specific pathogen free (SPF) barrier facilities strictly protected from their wild conspecifics. For many years the microbial starting point for many rodent breeding colonies has been the Altered Schaedler Flora (ASF) or variants thereof. ASF consists of eight specific bacterial strains originating from conventional laboratory mice from the 1960’s and 1970’s, and it is therefore considered mouse-specific^11,12^. In addition to this, laboratory rodents are exposed to microbes deriving from human staff in the facility, at least if bedding, food, and other materials are sterilized before introduced to the facility. Evolutionary adaptation to the host environment may drive formation of mouse-specific species and strains originally derived from humans, as human bacteria are still known to colonize the mouse gut far better than environmental bacteria^13^. It is known from transplantation of a zebrafish microbiota to mice that the microbiota composition in the recipient mice rather reassembles into one more similar to the recipient species than to the donor species, while the original endogenous balance between Proteobacteria and Firmicutes remained more or less preserved between zebrafish and mice^14^. So, despite core microbial^15^ and functional^16^ similarities, HM and laboratory mouse microbiota (MM) are clearly different from one another^10,15^, as well as the laboratory mouse microbiota is very different from the microbiota of pet shop and feral mice^17,18^.

In a range of animal models, single species of key bacteria are important for the expression of the model and some of these are also direct targets of intervention^19^. The CD4 surface molecule is expressed on all helper (Th) and regulatory T cells (Tregs)^20^, while FOXP3 is generally considered a specific marker of Tregs^21,22^. The surface receptor CD8 is classically considered a marker of cytotoxic T cells^23^, but it can also be expressed on natural killer cells^24,25^, dendritic cells^26^ and Tregs^27^.

The Gram positive, clostridial species, *Faecalibacterium prausnitzii*^28^, is in humans among the most abundant members of the gut microbiota constituting in the range of 2–5% of the total bacterial community^29,30^, and it is one of the key inducers of human FOXP3^+^ Tregs, some of which are known to be CD4^+^CD8^+^ ^31^. It has a clear probiotic potential, as shown by its ability to alleviate symptoms in the 2,4,6-trinitrobenzenesulphonic acid (TNBS) colitis model in mice^32–34^. Also, a high abundance of *Bifidobacterium* species is strongly correlated to low levels of inflammation in mice and it is therefore a commonly applied probiotic, e.g. in relation to colitis^35–39^. Although *F. prausnitzii* has been listed as one of the top 20 core bacterial genera of the mouse^16^ and is able to colonize the murine gut^40,41^, it appears totally absent in some mouse colonies^15^. *Bifidobacterium* spp. are also absent in many laboratory mice and at least much less abundant in mice than in humans^15^, and accordingly it is not listed as one of the top 20 core genera of mice^16^. Another example is *Akkermansia muciniphila*, which is a highly relevant target within type 1 diabetes^41,42^. This bacterium is, however, more common in commercial mouse colonies and therefore probably more easily colonizes mice^15^. Previously, Kibe *et al*. showed in 2005 that Actinobacteria were not transferred by HM transfer to mice, but it is unclear whether these Actinobacteria contained *Bifidobacterium* spp.^43^. At that time *Faecalibacterium* spp. were probably included in *Clostridium* cluster IVa, and these were transferred from human to mice in the studies by Kibe *et al*., but this cluster also contains other species. Interestingly, Kibe *et al*. observed Verrucomicrobia in the mice in spite of its absence in the human donor^43^, and these are most likely *Akkermansia muciniphila* as this is the only Verrucomicrobia species observed in mice until date^44^. As sequencing with better equipment has become deeper, it is today possible to describe the microbiota more precisely to a species level. In 2015 Wos-Oxley *et al*. showed that *Faecalibacterium* spp. appeared with a low abundance in HM transplanted ex-germ-free C57BL/6 (B6) mice, while this was not the case if transplanting to antibiotic-treated mice^45^.

Chung *et al*. showed how development of populations of CD4^+^ T-cells, CD8^+^ T-cells and dendritic cells as well as the expression of the antimicrobial regenerating islet-derived 3-gamma-peptide (REG3ɣ) was significantly impaired in HM-transplanted compared to MM-transplanted outbred Swiss Webster (SW) mice. Considering that there are substantial immunological differences between outbred and inbred mice, even between different inbred strains^46^, the immunological phenotype resulting from transplantation with HM is likely to be dependent on the recipient mouse strain or stock. The B6 mouse is one of the most widely applied inbred strains and various B6 substrains are used for linking metabolic syndrome and diabetic features with changes in the microbiota^47–50^ due to their susceptibility to diet-induced obesity^51,52^. Additionally, the B6 strain serves as the congenic background for a range of spontaneous and induced disease mutations, some of which are used for microbiome research, e.g. deficiencies of leptin^53^, apolipoprotein E^54^, and mucin 2 (MUC2) production^55^. Wos-Oxley *et al*. studied the colonization of HM transplanted to germ-free B6 mice, but they did not characterize the immune response in these mice^45^.

In the present study, we hypothesized that germ-free (GF) B6 mice transplanted with a xenogeneic HM would express a gut microbiota and immunological profile different from B6 mice with a syngeneic B6 MM. Similarly, we hypothesized that an allogeneic MM transferred from B6 to SW mice would result in bacterial colonization and immunological profiles different from those caused by a syngeneic MM transferred from B6 to B6.

## Results

**Figure 1:**
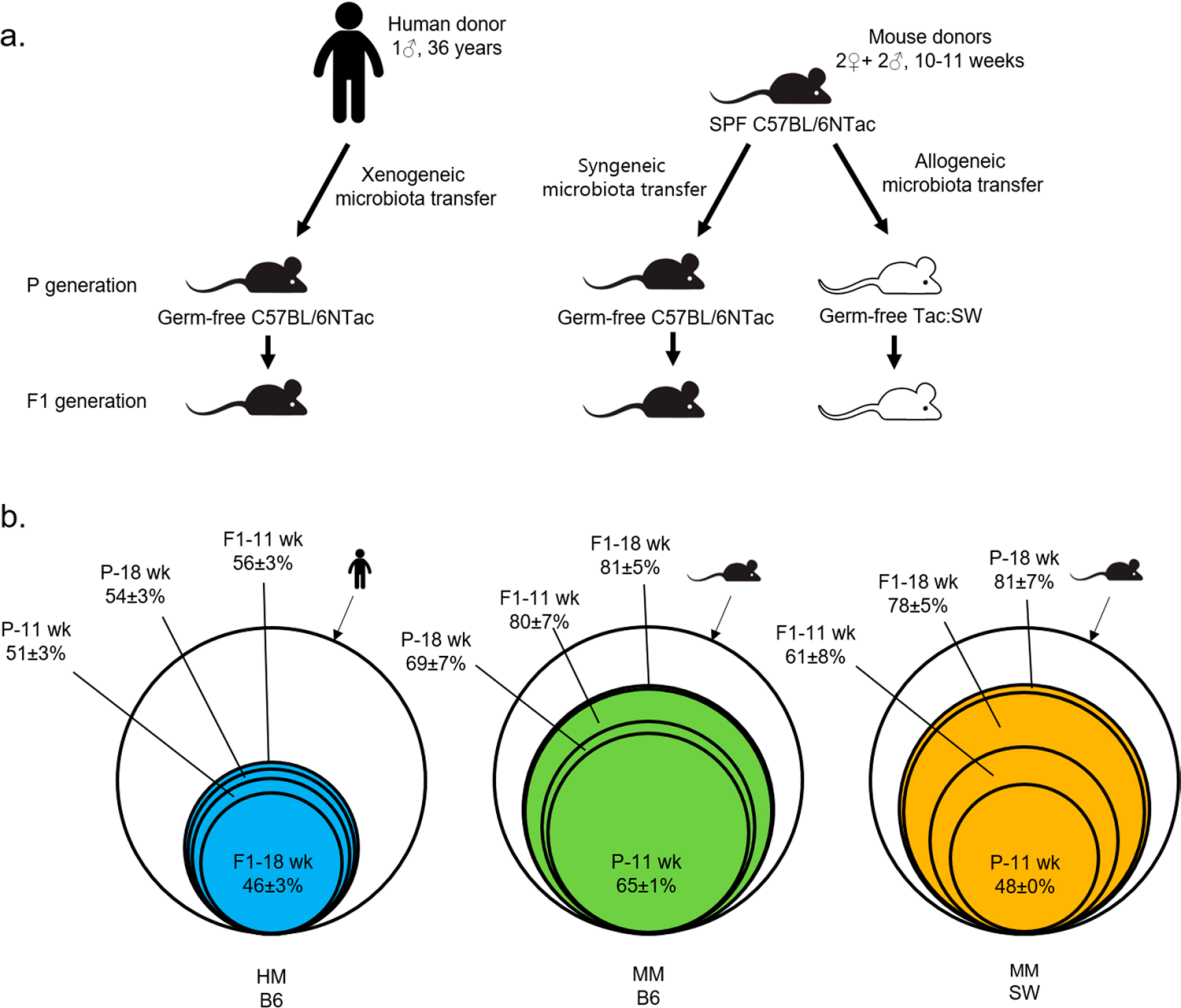
(**a**) Study design. n = 4♀ (P HM B6/MM B6/MM SW); 8♀, 12♂ (F1 HM B6); 14♀,12♂ (F1 MM B6); and 25♀,19♂ (F1 MM SW) (see Table 1). Germ-free B6 and SW mice were transplanted when six weeks old with HM or B6 MM and housed in gnotobiotic isolators. The transplanted P mice were bred and the offspring (F1) generation born with the microbiota (related to Table 1). (**b**) Colonization efficiency. The proportion of operational taxonomic units (OTUs) from the human and mouse donor samples that were detected in the recipient P and F1 mice is shown. The outermost non-filled circle represents the donor samples, i.e., 100%. Each smaller circle represents fecal samples sorted generation- and age-wise and with standard deviation. From left to right: HM donor and B6 recipients; B6 MM donor and B6 recipients; B6 MM donor and SW recipients. HM = human microbiota, MM = mouse microbiota, B6 = C57BL/6NTac, SW = Tac:SW (Swiss Webster), P = transplanted parent generation, F1 = offspring generation born with microbiota.

One human fecal donor microbiota (HM), obtained from a healthy, non-vegetarian, male, adult donor, and a pool of fecal microbiotas derived from two female and two male B6 donor mice (MM) were used as inoculates. The HM was transplanted into GF B6 mice, while the MM was transplanted into GF B6 as well as GF SW mice. In all groups, four female and two male mice were used as recipients (P; parental generation), which were then bred to produce three separate offspring F1 generations exposed to the introduced microbiota from birth (Fig. 1a; Table 1). We measured gene expression of selected markers of the innate and adaptive immune system as well as the intestinal barrier in gut tissue, cytokines and chemokines in plasma and assessed microbiota composition and colonization efficiency by means of bacterial 16S ribosomal RNA (rRNA) amplicon sequencing. The immunological targets were, based on literature and previous experience in our laboratory, chosen for previously being reported to be regulated by the gut microbiota.

**Table 1:** Study groups.

Group	Group size for microbiota characterization	Group size for gene expression in colon^a^	Group size for gene expression in ileum^a^	Group size for plasma measurements	Time of colonization (age)	Time of fecal sampling (age)	Time of colon/ileum tissue sampling (age)
HM-B6-P	4♀	4♀	NA	4♀	6 weeks	11 + 18 weeks	18 weeks
HM-B6-F1	8♀ + 12♂	8♀	8♀ + 10♂	8♀ + 12♂	Natural transmission from dam	11 + 18 weeks	18 weeks
MM-B6-P	4♀	4♀	NA	4♀	6 weeks	11 + 18 weeks	18 weeks
MM-B6-F1	14♀ + 12♂	14♀	10♀ + 10♂	14♀ + 12♂	Natural transmission from dam	11 + 18 weeks	18 weeks
MM-SW-P	4♀	NA	NA	NA	6 weeks	11 + 18 weeks	18 weeks
MM-SW-F1	25♀ + 19♂	NA	10♀ + 8♂	NA	Natural transmission from dam	11 + 18 weeks	18 weeks
RF (SPF) B6	NA	4♀	4♀	NA	NA	NA	12 weeks
GF B6	NA	3♀	3♀ + 4♂	4♀ + 4♂	NA	NA	6 weeks
GF SW	NA	NA	4♀ + 4♂	NA	NA	NA	6 weeks

Germ-free (GF) human microbiota (HM)-colonized C57BL/6NTac (B6) and mouse microbiota (MM)-colonized B6 and Tac:SW (SW) were bred and the parent (P) and offspring (F1) generations subjected to microbiota characterization by 16 S rRNA amplicon sequencing and gene expression profiling of colon and ileum. ^a^Samples for gene expression were randomly selected from the full cohort of samples shown in column “Group size (microbiota characterization)”.

### MM was most efficiently transferred, while several key bacteria were lost after HM transplantation

**Figure 2:**
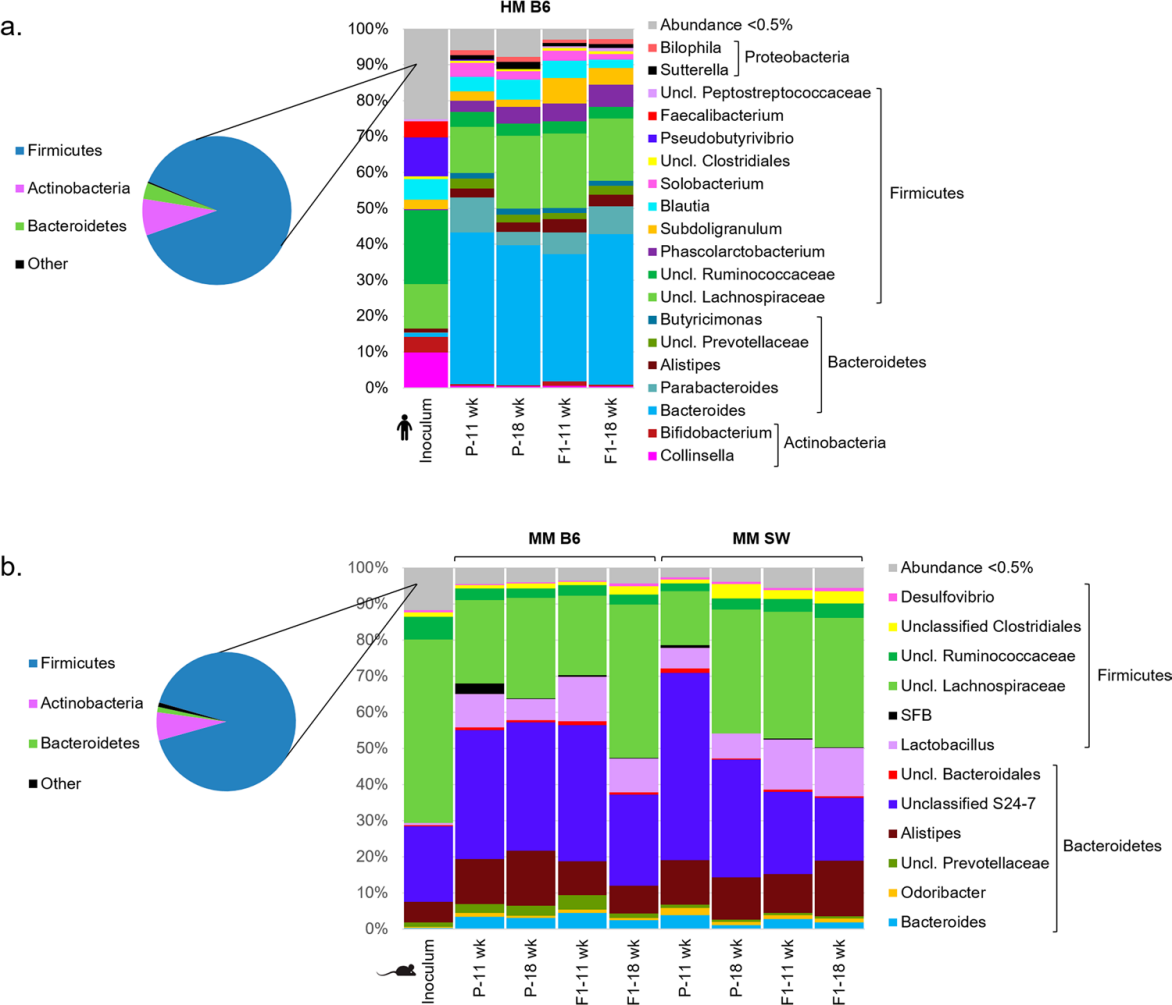
Gut microbiota composition of HM and MM mice. n = 4♀ (P HM B6/MM B6/MM SW); 8♀, 12♂ (F1 HM B6); 14♀,12♂ (F1 MM B6); and 25♀,19♂ (F1 MM SW)(see Table 1). (**a**) Relative abundance chart showing genus level composition of fecal samples from B6 mice (11 and 18 wk of age) colonized with HM from a male, human donor. (**b**) Relative abundance chart showing genus level composition of fecal samples from B6 and SW mice (11 and 18 wk of age) colonized with MM from a pool of male (n = 2) and female (n = 2) B6 donors. Genera with abundance below 0.5% were aggregated into a single group in a. and b. Composition on phylum level is shown in the pie charts. HM = human microbiota, MM = mouse microbiota, B6 = C57BL/6NTac, SW = Tac:SW (Swiss Webster), P = transplanted parent generation, F1 = offspring generation born with the microbiota.

Colonization of the MM from B6 mice was largely equally efficient in GF B6 and GF SW mice (Fig. 1b). At 18 wk of age, the F1 B6 harbored 81 ± 5% of the MM inoculum OTUs, while the F1 SW mice harbored 78 ± 5% (p = 0.79). HM-colonized mice reached 56 ± 3% as the highest representation of OTUs from the HM inoculum in F1 mice at 11 wk of age and were significantly different from B6 and SW mice at the same age with respect to establishment of the introduced bacteria (p < 0.001; Fig. 1b). HM recipients had similar relative abundance profiles of P and F1 mice at different ages, while there was a marked compositional difference between the original HM inoculum and the feces of the recipients (Fig. 2a; Supplementary Table S1). Several genera, including ones taxonomically assigned to *Pseudobutyrivibrio*, *Faecalibacterium*, *Bifidobacterium* and *Shuttleworthia* were almost or completely lost in the HM mice (Fig. 2a). *Bacteroides*, on the other hand, increased dramatically in the HM mice from 1% in the inoculum to 39 ± 11% in recipient mice. The microbiotas of the MM colonized mice were more similar to the original inoculum than what was observed for the HM colonized mice. However, there were some minor differences between the microbiotas of the MM recipient mice and the MM inoculum, including a decrease in *Lachnospiraceae* family such as *Shuttleworthia*, and a pronounced increase in *Lactobacillus* in both the B6 and SW recipients after transplantation (Fig. 2b; Supplementary Tables S2 and S3). Body weight did not differ between B6 mice colonized with HM and MM (Supplementary Fig. S1).

### Transplantation with MM or HM resulted in different microbial diversity in the recipient mice

**Figure 3:**
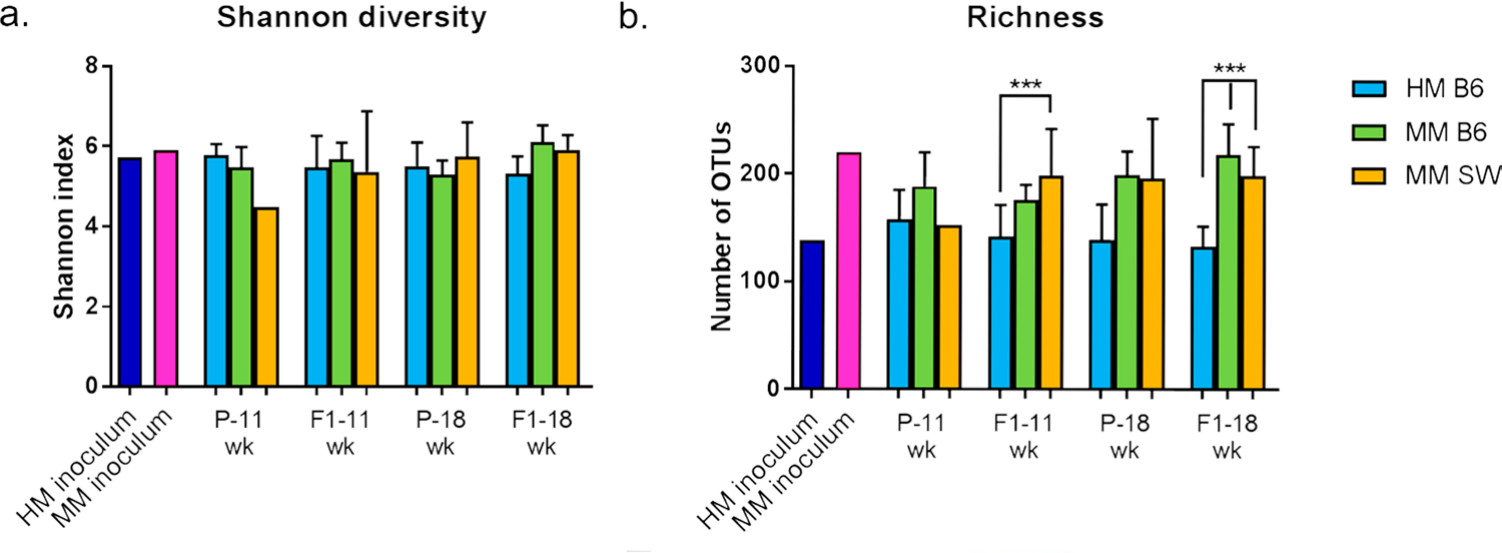
Alpha diversity of HM and MM fecal samples. n = 4♀ (P HM B6/MM B6/MM SW); 8♀, 12♂ (F1 HM B6); 14♀,12♂ (F1 MM B6); and 25♀,19♂ (F1 MM SW)(see Table 1) (**a**) Shannon diversity and (**b**) Richness of fecal samples from B6 mice colonized with HM or MM and from SW mice colonized with B6 MM. Samples were collected when P and F1 were 11 and 18 weeks old. ANOVA with Tukey’s comparisons, bars are SD. ***p < 0.001. HM = human microbiota, MM = mouse microbiota, B6 = C57BL/6NTac, SW = Tac:SW (Swiss Webster), P = transplanted parent generation, F1 = offspring generation born with the microbiota.

**Figure 4:**
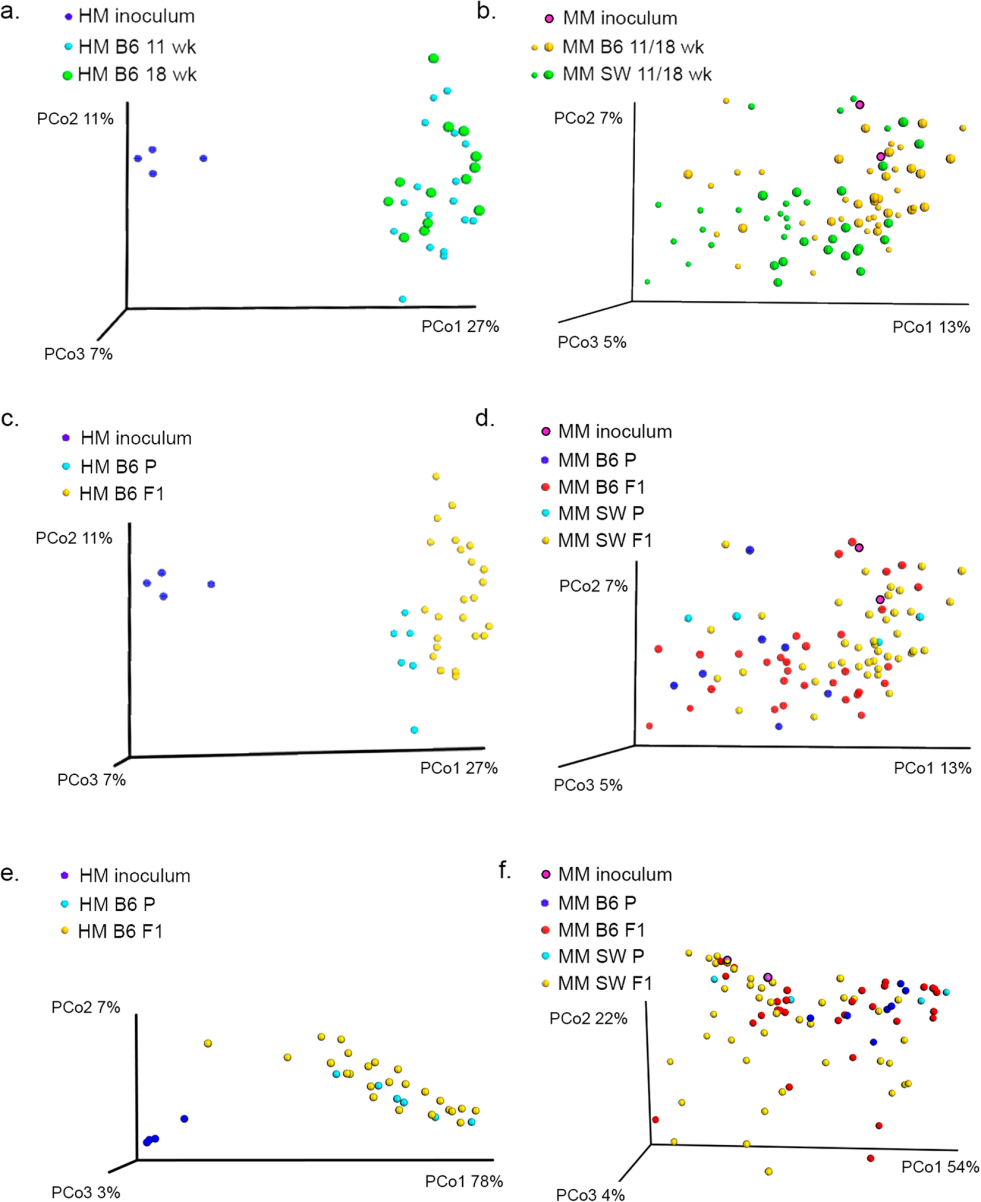
Beta diversity of HM and MM fecal samples. (**a-d**) PCoA plots based on unweighted UniFrac distance matrices. The inoculum samples are PCR replicates of a single inoculum sample. HM samples are very distinct from the HM inoculum (**a+c**). MM samples cluster together with MM inoculum (**b+d**). Coloring of samples according to P to F1 generation revealed generation-wise drift in the HM mice (**c**), which was absent in the MM mice (**d**) Large spheres=18 wk; small spheres =11 wk old mice. PCoA plots based on weighted UniFrac distance matrices revealed comparable patterns to the unweighted analyses, except that the generation-wise separation disappeared in the HM group (**e+f**). HM = human microbiota, MM = mouse microbiota, B6 = C57BL/6NTac, SW = Tac:SW (Swiss Webster), P = transplanted parent generation, F1 = offspring generation born with the microbiota.

Alpha diversity as assessed by the Shannon index remained stable over time and did not differ between HM- and MM-transplanted mice (Fig. 3a). Richness (total number of OTUs) was higher in the MM inoculum, which was pooled from four mice, compared to HM inoculum. This pattern was reflected in the recipients, as richness was significantly higher in MM SW F1 mice aged 11 wk, and in MM B6 and SW F1 mice aged 18 wk compared to HM-transplanted mice of the same age (Fig. 3b). Unweighted and unweighted UniFrac distance matrices visualized in 3D PCoA plots revealed a strong separation between the original HM inoculum and the mice transplanted with HM, as the mean Unifrac distance between HM inoculum and HM mice was significantly larger than the distance between MM inoculum and B6 and SW mice, respectively (unweighted: p < 0.0001 (Fig. 4a+c); weighted: p < 0.001 (Fig. 4e).The MM B6 and MM SW recipients appeared to cluster together but were nonetheless distinct from each other in ANOSIM tests (unweighted: p = 0.003, R = 0.19 at 11 wk, and p = 0.001, R = 0.26 at 18 wk; Fig. 4b+d; weighted: p = 0.009, R = 0.16 at 11 wk and p = 0.09, R = 0.05 at 18 wk; Fig. 4f). The mean UniFrac distance from the MM B6 mice and MM SW mice, respectively, to the MM inoculum was not different. In the unweighted analysis, HM P mice clustered separately from HM F1 samples (p = 0.01, R = 0.43; Fig. 4c), while this was not the case for the MM P and F1 samples (Fig. 4d). There was no generation-wise clustering revealed from the weighted analyses (Fig. 4e+f). Altogether, the unweighted and weighted analyses indicate that qualitative as well as quantitative features of the microbial communities play a role in discriminating the HM samples according to inoculum/recipients, whereas separation according to P or F1 generation is mostly characterized by presence/absence of rare taxa (Fig. 4c+e).

### Mice colonized with human microbiota had low expression levels of immune-related genes in the gut

**Figure 5:**
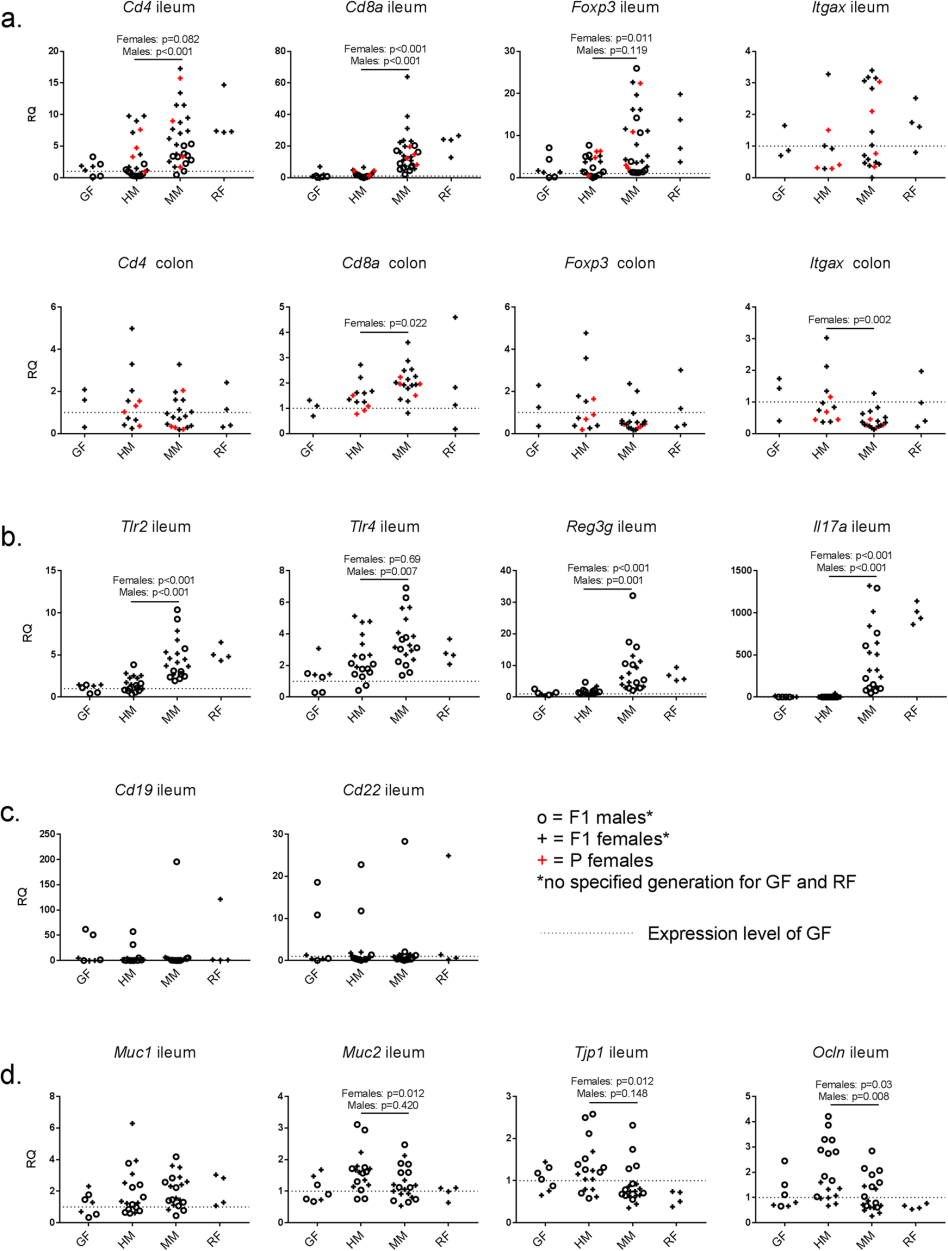
Relative quantification of genes measured in intestinal tissue. Ileum measurements: n = 8♀,10 ♂ (HM); 10♀,10 ♂ (MM); 3♀,4♂ (GF B6); 4♀,4 ♂ (GF SW) and 4♀ (RF) Colon measurements: n = 4♀ (P HM/MM/RF); 3♀ (P GF); 8♀ (F1 HM); 14♀ (F1 MM)(see Table 1) **a**. Genes encoding markers for cytotoxic T cells, T helper cells, regulatory T cells and dendritic cells (*Cd4*, *Cd8a*, *Foxp3, Itgax)* measured in ileum and colon, and **b**. toll-like receptors, REG3ɣ and IL-17a (*Tlr2*, *Tlr4*, *Reg3g*, *Il17a*), and **c**. B cells (*Cd19*, *Cd22*), and **d**. mucins and tight junction complex proteins occludin and tight junction protein 1 (*Muc1*, *Muc2*, *Ocln*, *Tjp1*) measured in ileum of B6 F1 mice with HM or MM, and in GF and RF barrier-raised B6 mice. Student’s t-test between HM and MM were performed separately for male and female mice. P female mice were included for measurements of *Cd4*, *Cd8a*, *Foxp3, Itgax* and were not statistically different from F1 female mice. Only female mice were included for the colon measurements. GF = germ-free, RF = Restricted Flora (=specific pathogen free with opportunists excluded), HM = human microbiota, MM = mouse microbiota, B6 = C57BL/6NTac, P = transplanted parent generation, F1 = offspring generation born with the microbiota, RQ = relative quantification.

We measured gene expression of *Cd4*, *Cd8a*, *Foxp3*, and *Itgax* responsible for production of T and dendritic cell (DC) markers CD4, CD8a, forkhead box P3 (FOXP3) and alpha x integrin (Itgax or CD11c) in ileum and colon tissue from P and F1 HM and MM B6 mice at 18 wk of age (Fig. 5a). For comparison, GF and Restricted Flora™ (RF™; an SPF microbiota) B6 mice corresponding to the MM donors were included as reference. There were no differences between P and F1 mice with respect to expression of the four genes that were analyzed in both generations, i.e., *Cd4*, *Cd8a*, *Foxp3*, and *Itgax*, and the two generations were subsequently analyzed together. On the other hand, there were significant sex effects in the expression of *Cd4*, *Foxp3*, *Muc2*, *Tjp1* and *Tlr4*, and accordingly we analyzed males and females separately for all targets. In ileum, *Cd8a* expression was lower in HM than in MM mice for both sexes (p ≤ 0.001), *Cd4* expression was lower in HM males, and *Foxp3* expression was lower in HM female mice (Fig. 5a). In colon, only *Cd8a* was lower in HM female mice compared to MM mice (p = 0.022), whereas *Itgax* expression was higher in HM mice (p = 0.002; Fig. 5a). Expression levels of genes coding for toll-like receptors (TLR) 2 and 4, REG3ɣ and the pro-inflammatory interleukin 17a (IL-17a), i.e., *Tlr2*, *Tlr4*, *Reg3g*, and *Il17a*, were also measured in ileum (Fig. 5b). *Tlr2* was less expressed in HM mice compared to MM in both sexes (p < 0.001). *Tlr4* was less expressed in HM males (p = 0.007), but not in females. *Reg3g* was lower in HM compared to MM in both sexes (p ≤ 0.001), and *Il17a* was hardly or not at all expressed in HM mice, but was high in male and female MM mice (p < 0.001). The genes *Cd4*, *Cd8a*, *Foxp3*, *Tlr2*, *Tlr4*, *Reg3g* and Il17a were not differentially expressed in GF and RF^TM^ reference mice and HM and MM mice (Fig. 5a+b). Expression of Cd19 and Cd22, genes encoding pan-B cell marker CD19 and activated B cell marker CD22, measured in ileum, were generally very low for HM, MM, GF and RFTM and not different between the groups, although with few extreme outliers in all groups (Fig. 5c). Expression of *Muc1*, *Muc2*, *Tjp1*, and *Ocln* responsible for production of barrier-related proteins mucin 1 (MUC1), MUC2, tight junction protein 1 (Tjp1, or ZO-1), and occludin (Ocln) was additionally assessed in ileum (Fig. 5d). *Muc2* and *Tjp1* were expressed less in MM than in HM for females, but not for males (p = 0.012 for both genes). Expression of *Ocln* was lower in MM than in HM for both sexes (p = 0.03 for females and p = 0.008 for males; Fig. 5d). The GF and RF^TM^ reference expression levels of *Muc1*, *Muc2*, *Tjp1*, and *Ocln* were not significantly different when compared to each other or to HM and MM mice (Fig. 5d).

**Figure 6:**
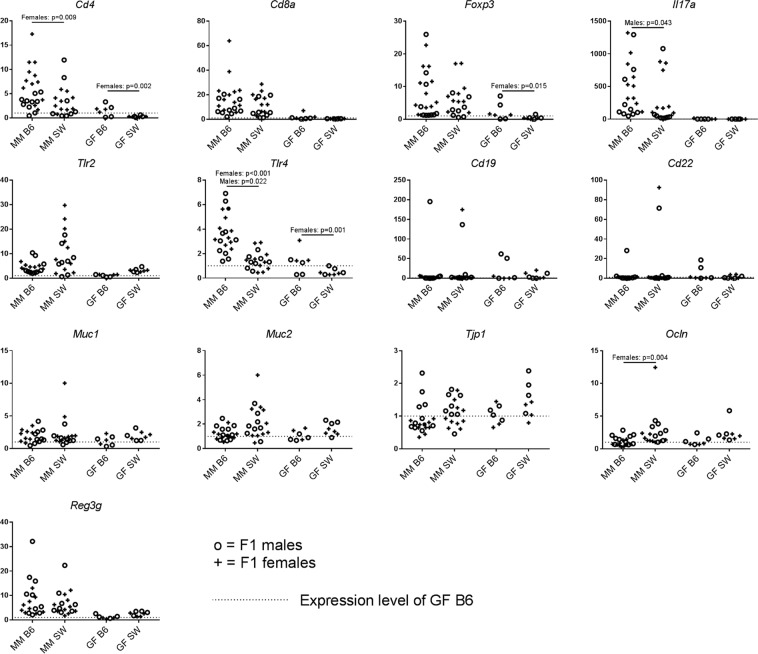
Relative quantification of genes measured in ileum of germ-free and mouse microbiota-colonized B6 and SW mice. n = 10♀, 10♂ (MM B6); 10♀,8♂ (MM SW); 4♀, 4♂ (GF B6) and 3♀, 4♂ (GF B6) (see Table 1). Genes encoding markers for cytotoxic T cells, T helper cells, regulatory T cells and dendritic cells (*Cd4*, *Cd8a*, *Foxp3, Itgax)*, toll-like receptors, REG3ɣ and IL-17a (*Tlr2*, *Tlr4*, *Reg3g*, *Il17a*), B cells (*Cd19*, *Cd22*), and mucins and tight junction complex proteins occludin and tight junction protein 1 (*Muc1*, *Muc2*, *Ocln*, *Tjp1*) were measured in ileum of F1 SW and B6 mice with a B6 MM at 18 wk of age, and in GF B6 and SW mice at 6 wk of age. ANOVA with Tukey’s multiple comparisons post hoc test performed separately for males and females. GF = germ-free, MM = mouse microbiota, B6 = C57BL/6NTac, SW = Tac:SW (Swiss Webster) P = transplanted parent generation, F1 = offspring generation born with the microbiota, RQ = relative quantification.

Gene expressions in MM and GF B6 mice were compared to MM and GF SW mice to assess if expression levels were dependent on mouse genetics. *Cd4* and *Tlr4* had a higher expression in female MM B6 mice compared to MM female SW mice (p < 0.009 and p < 0.001; Fig. 6). Likewise, expression of *Cd4* and *Tlr4* were higher in female GF B6 mice compared to GF female SW mice (p < 0.002 and p = 0.001; Fig. 6). Expression of *Ocln* was lower in MM B6 females compared to MM SW female (p = 0.04), whereas there was no difference between GF B6 and SW mice for this target. For male mice, expression levels of *Tlr4* were higher in MM B6 compared to SW (p = 0.022; Fig. 6), but there was no difference between GF male B6 and SW. Similarly, *Il17a* had higher expression levels in male MM B6 males compared to SW (p = 0.043), whereas *Il17a* was hardly or not expressed in neither GF B6 nor GF SW mice. Expression of *Foxp3* was higher in GF B6 females compared to BF SW females (p = 0.015; Fig. 6).We performed quantitative linear regression between the absolute, rarefied abundance of OTUs in MM and HM mice and the gene expression ΔC_T_ values, but no associations were observed (data not shown).

### Human and murine microbiota have disparate effects on systemic immunity

**Figure 7:**
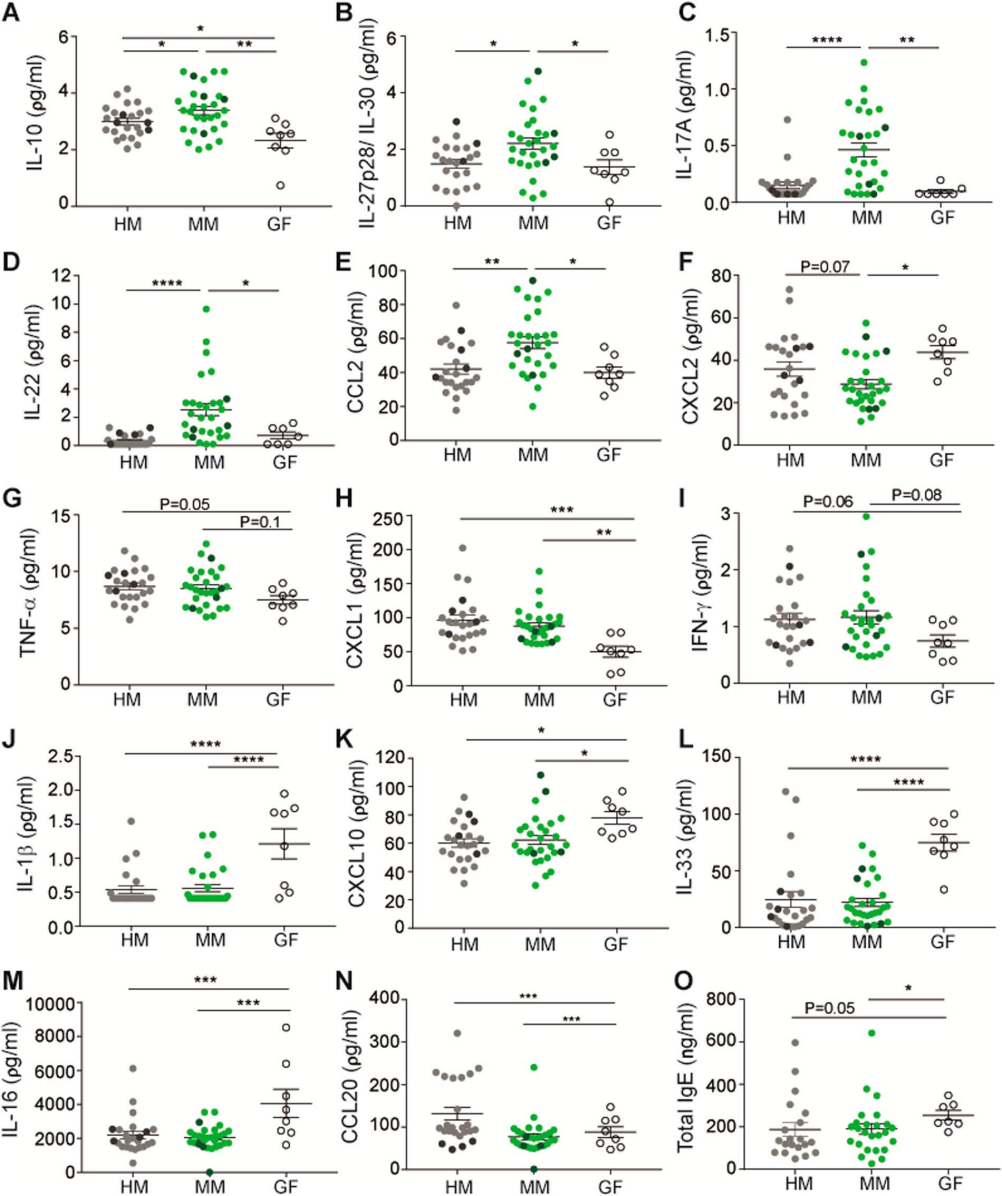
Systemic levels of cytokines, chemokines and total IgE in germ-free, mouse- and human microbiota-colonized B6 mice. n = 12♀,12♂ (HM) and 18♀,12♂ (MM) (see Table 1). 29 cytokines and chemokines (**A**-**N**) as well as total IgE levels (**O**) were measured in plasma of human microbiota-colonized (HM) parent (dark grey) and F1 mice (light grey), mouse-microbiota colonized (MM) parent (dark green) and F1 mice (light green) at 18 wk of age, and germ-free (GF, empty circles) mice at 6 wk of age. Only plots with significant differences or p < 0.1 are shown. Mean and SEM are shown. * indicates p < 0.05, ** indicate p < 0.01, *** indicate p < 0.001, **** indicate p < 0.0001. F1 = offspring generation born with the microbiota, B6 = C57BL/6NTac.

To investigate whether the human and murine microbiota profiles also diverged in their ability to stimulate the systemic immune response of the host, 29 cytokines and IgE levels were measured in plasma on a multiplex panel. There were no differences between the groups in IL-5, IL-15, CCL3, IL-17C, IL-17E/IL25, and IL-2. Four of the cytokines (IL-10, IL-27p28/ IL-30, IL-17A, and IL-22) were significantly higher in MM mice compared to HM and GF mice (Fig. 7A–D). Also, the chemokine CCL2 was significantly higher in the MM mice (Fig. 7E), whereas the neutrophil recruiting chemokine CXCL2 was significantly lower in MM mice compared to both HM and GF mice (Fig. 7F). Colonized mice produced higher levels of IFN-γ, TNF-α, and CXCL1 (Fig. 7G–I) and lower levels of IL-1β, IL-33, IL-16, CXCL10, and CCL20 compared to GF mice (Fig. 7J–N). However, a complete microbiota was not necessary as the MM and HM microbiota were equally able to induce a cytokine or chemokine response different from that of the GF mice. The serum IgE levels were highest in the GF mice and no difference was evident between the MM and HM mice (Fig. 7O).

## Discussion

Transplantation of a complex HM into mice revealed that some of the key anti-inflammatory bacteria from humans, *Bifidobacterium* and *F. prausnitzii*, were not well established in the B6 mouse gut, which is in line with previous observations in SW mice^56^, and probably also in line with older studies with a lower taxonomic resolution^43^. However, we cannot exclude that a strict anaerobe like *F. prausnitzii* could have been nonviable in the inoculum. Also in line with our observations, both Kibe *et al*.^43^ and Wos-Oxley *et al*.^45^ observed overabundance of *Bacteroides* spp. in HM transplanted mice. *Itgax*, which is a membrane protein expressed on dendritic cells as well as on myeloid cells such as macrophages, monocytes and neutrophils^57,58^, showed increased expression in the colon of HM mice, which may reflect an inflammatory response against human-derived bacteria. Lower abundances in recipient mice, as for example observed for *Lachnospiraceae*, do not reflect that these taxa were lost by handling of the inoculum. On the other hand, increased abundances of lactobacilli, as seen in our study, are a common observation in microbiota transfers in mice^59^, which could be due to the ability of lactobacilli to colonize the non-glandular part of the stomach^60^.

The richness of the MM inoculum was higher than that of the HM inoculum. Mice raised in commercial SPF barrier facilities have been shown to lack or have a low abundance of bacteria consistently observed in wild mice^18^, which may be due to the extensive cleaning procedures aimed at the exclusion of pathogens^61^. In our study, we may artificially have increased the richness by pooling feces from four individual mice for the MM donor material compared to the single human donor sample. In contrast, the use of a single human donor may limit the interpretation of the study, although the gut microbiota of healthy Danes cluster quite narrowly and also cluster with Canadians and Italians^62^, who like Danes eat a diet of pasta, bread, vegetables and varying amounts of pork, beef, chicken and fish. Our donor is quite close to the average of a Danish healthy microbiota harboring the core species^62^, although he has a lower abundance of Proteobacteria. Using a Japanese, Austrian or rural Tanzanian donor would obviously have been different^62^.

Our gene expression data confirm that CD4 and CD8 positive cells are reduced in numbers in HM transplanted mice^63^. SPF mice in general have fewer CD8 positive cells compared to pet shop mice, which could be due to differences in virus status^17^. Antibiotic treatment reduces gene expressions of acute inflammation^64^. It does not seem to have acute or long term effects on expression on the level of CD4, CD8 and FoxP3 positive cells^65^, but it may reduce the activation of CD4 positive T cells in the tonsils^66^. MM transplantation from healthy to IBD induced mice increases levels of FoxP3 positive cells^67^. So, generally the levels of CD4, CD8 and FoxP3 positive cells seems to be mostly correlated to the initial stimulation of the immune system, which fits well with the observations of us and Chung *et al*.^63^ that MM is a strong stimulant and HM is a weak stimulant. The lack of *Bifidobacterium* and *F. prausnitzii* in HM mice may have influenced the lower expression of T cell marker genes *Cd8a*, *Cd4*, and *Foxp3*, and of the gene for the antimicrobial peptide *Reg3g*. As *Tlr2* and *Tlr4*, both of which are known to be influenced by microbiota colonization^68,69^, also displayed lower expression in HM compared to MM mice, reduced activation of TLRs may also have had an impact^70,71^. It is known that TLR recognition of some commensals, e.g. *Lactobacillales*, also requires host-adapted microbe-associated molecular patterns^72,73^. Mice deficient of the TLR downstream adaptor protein MyD88^74^ display significantly decreased populations of CD8a^+^ intestinal lymphocytes, however only in the intra-epithelial lymphocyte compartment and not in the lamina propria^75^. Therefore, low TLR stimulation cannot be the only explanation of the observed differences. The discovery of CD8^+^FOXP3^+^ regulatory T cells^76^ is interesting, because one explanation for the low expression of *Cd4*, *Cd8* and *Foxp3* may be that the mice have low numbers of both CD4^+^FOXP3^+^ and CD8^+^FOXP3^+^ Tregs. We have previously shown that contact between the gut immune system and bacteria present in a mouse model of colitis could increase the number of CD8^+^FOXP3^+^ Tregs^77^, and, therefore, the observed immune stimulation in the MM mice may have been elicited by bacterial species with a higher affinity to mice. Ileal expression of *Il17a* in ileum and systemic IL-17A and IL-22 were very low or even absent in HM but high in MM mice. In another study, HM mice compared to MM mice exhibited significantly lower Th1/Th17-dependent responses to infection with *Campylobacter jejuni*^78^. Such observations may reflect that MM mice harbor Segmented Filamentous Bacteria (SFB), which are known as the most potent inducers of murine Th17 cells, i.e. CD4^+^ Th cells producing IL-17 and IL-22^79^, while other bacteria fail to efficiently stimulate intestinal T cell responses in the same way^80^. Human-derived *Bifidobacterium adolescentis* are also capable of inducing a Th17 response in mice, however without provoking inflammation^81^. It should be noted that we did not find any correlation in the mice between *Il17* expression and the abundance of SFB. It cannot be excluded that monitoring gene expressions at another time point could have given another result. We additionally found that the human microbiota was less capable of inducing systemic anti-inflammatory cytokines IL-10 and IL-27p28/ IL-30 and CCL2, a chemokine involved in downregulation of proinflammatory responses^82^. These findings are in line with the finding that MM mice produced less of the proinflammatory chemokine CXCL2 and altogether our results demonstrate that the host origin of a complex, transplanted microbiota influences the adaptive immune system locally as well as systemically. Serum IgE levels are known to be downregulated by the gut microbiota in mice transplanted with an SPF microbiota^83^, and we show here that a human microbiota has capacity to inhibit IgE systemically. Our data do not indicate that function of the intestinal barrier requires a host-specific microbiota.

Notably, animals transplanted at six weeks of age (P generation) developed an immunological gene expression phenotype at 18 weeks of age, which was similar to that of mice exposed to the microbiota from birth (F1 generation). A GF period in early life has previously been shown to dramatically affect immunological shaping later in life^84^, and the lack of difference between ex-GF transplanted P mice and F1 born with the microbiota observed in our current study could be related to factors such as the age at measurement, the composition of the transplanted microbiota, the marker genes we selected, and, importantly, the low number of mice we had in the P groups. However, that the gene expression phenotype seems not to differ between the transplanted and offspring mice is important when designing future transplantation studies as it indicates that larger group sizes of mice with a transplanted microbiota may be achieved more easily by using the offspring generation of transplanted mice without altering the phenotype compared to the transplanted mice.

Establishment of a transplanted B6 microbiota syngeneic to B6 recipients and allogeneic to SW recipients was equally efficient and there were only minor differences between B6 and SW microbiota compositions. This is not surprising, even though inbred mouse colonies mostly have a unique microbiota^85^. However, the lower inter-individual variation in a group of inbred mice and the differences in bacterial abundances between strains is most likely due to the breeding system in which breeding females are always close relatives originating from the same stem mother and housed in a very standardized and stable environment^86^. Colonization depends on host immune function, genetics and environmental exposure to microbes with genetics estimated to account for less than 20% of the microbiota variation^87^, and the microbiota can easily be swabbed between two inbred strains by fecal matter transplantation^88^. The B6 and SW mice were housed in open-top cages in the same isolator, a practice allowing sharing of microbes between cages, which is likely to have facilitated the similarity of the microbiotas between groups. On the other hand, housing the groups in distinct isolators also poses a risk of microbiota segregation in each isolator, which could artificially have exaggerated the effect of genetic background. When designing the study, we specifically chose to house the B6 and SW MM mice in the same isolator to be able to assess the true effect of genetics and avoid what we considered a bigger confounder, namely housing in separate isolators. Gene expression data of MM B6 and MM SW mice revealed minor genetic background effects in expression of *Cd4* (only significant in female mice) and *Tlr4*, which were also observed in GF mice^87^. *Tlr4* has been shown to differ between mouse strains^89^, which may lead to differences in response to bacterial infection^46^. That expression of *Cd4* and *FoxP3* was only significantly different between B6 and SW in the females may be explained by the fact that the intracellular progesterone receptor^90^ and the estrogen receptor alpha both regulates T cells. Both *Tlr2* and *Tlr4* expression is known to differ between sexes^91^, but in our study we found significant differences between MM B6 and MM SW mice for both sexes.

In future studies it would be relevant to work with the diet as a means to improve colonization of a HM in mice, as compared to the omnivore humans mice are fed a vegetarian, low-fat chow diet. Kibe *et al*.^43^ relate the overabundance of *Bacteroides* spp. to chow feeding, and in the same way Wos-Oxley *et al*.^45^ relate the reduced abundance of *F. prausnitzii* to feeding. Another future study could be the modification of receptors and signaling pathways in the immune system of the recipient mice. Finally, even though commercial laboratory mice are sold as virus free, this status is based upon a limited number of serological assays^92^, and it is not unreasonable to assume that both MM and HM contains species specific immune stimulating enteroviruses, which are not transferred in a xenogeneic microbiota transfer.

In conclusion, we have shown that important immune-regulating bacteria are lost when transplanting microbiota from humans to B6 mice and that the established human microbiota is a weaker stimulator of the murine immune system compared to bacterial communities derived from mice. Substantial differences in expression of immune-related intestinal genes as well as cytokines and chemokines in plasma were observed between mice transplanted with a HM and an MM, respectively, while transplantation of an allogeneic as opposed to a syngeneic microbiota between mice did not reduce the number of established bacteria.

## Materials and Methods

### Microbiota colonization and housing

Two groups of four female and two male GF C57BL/6NTac (Taconic Biosciences, Germantown, US), and four female and two male GF Tac:SW (Taconic Biosciences) were colonized by oral gavage (50 µl/mouse) with gut microbiota when 6 weeks (wk) old. B6 mice were colonized with either HM or MM, while SW mice were colonized with MM (Fig. 1a). The donor microbiotas (inoculates) were prepared by homogenizing freshly voided human feces or freshly harvested mouse distal colonic contents in sterile 25% glycerol (Merck Millipore) subsequently frozen at −80 °C until use. To align conditions between the HM and MM inoculum, we chose not to harvest material for the MM inoculum under anaerobic conditions, as this was not possible to achieve for the HM. The MM donors were two female (11 wk of age) and two male (10 wk of age) C57BL/6NTac mice (Taconic Biosciences, Lille Skensved, Denmark) with Taconic’s Restricted Flora (RF) health standard, which is an opportunist and specific pathogen free microbiota. The HM donor was a healthy, non-vegetarian 36 years old Caucasian male with a body mass index within the normal range (18.5–24.9), and with no history of antibiotic treatment for at least 12 months before submitting the fecal sample to the study. The human donor was serologically screened negative for Hepatitis A, B and C, *Treponema pallidum*, HIV-1 and HIV-2, and the human fecal sample was screened negative for *Helicobacter pylori*, *Salmonella sp*., *Shigella sp*., *Yersinia sp*., *Campylobacter sp*., *Clostridium difficile*, *Aeromonas sp*., *Plesiomona*s *sp*., *Vibrio sp*., pathogenic *E. Coli* strains, *Cryptosporidium*, *Giardia*, helminths and rotavirus, and additionally for the presence of *Proteus sp*., *Klebsiella oxytoca*, *Klebsiella pneumoniae*, *Citrobacter rodentium*, *Staphyloccous aureus* and *Pseudomonas aeruginosa*. The colonization procedure was aseptically performed in a biosafety cabinet decontaminated with 1:5:1 Clidox-S (Indulab). Donor material stored at −80 °C without glycerol was used for the downstream analyses. The colonized mice (referred to as P for parent mice) were aseptically transferred to two sterile flexible film isolators (CBC, Madison, WI, US) designated for each of the two microbiotas and socially housed in Eurostandard Type II L polycarbonate open-top cages (Tecniplast, Varese, Italy). The isolators were tested sterile for three consecutive weeks by aerobic and anaerobic culturing of bacteria and fungi before animals and materials were introduced. The isolator had approximately eight air changes/hour, light/dark cycle was 12/12 hours, the temperature 20–23 °C, and the ambient relative humidity was in the range of 45–65%. Bedding was JELUXYL HW 300/500 (JELU WERK, Rosenberg, Germany), nesting material Soft Paper Wool (Brogaarden, Lynge, Denmark) and gnawing blocks Aspen size S (Tapvei, Harjumaa, Estonia). The diet for all mice in the study, parents and offspring, was *ad libitum* ssniff M-Z Low-Phytoestrogen V1154-3 breeding diet for mice (ssniff Spezialdiäten GmbH, Kiel, Germany), and drinking water was autoclaved bottled tap water. All supplies were introduced aseptically to the isolators via autoclaved cylinders. After one week of acclimatization, the P mice were bred resulting in four litters per group (referred to as F1 mice) distributed on 8 female and 12 male HM B6 mice, 15 female and 12 male MM B6 mice, and 25 female and 19 male MM SW mice (Fig. 1a, Table 1). After weaning at 4 wk of age, the pups were housed two-three mice/cage. Fecal samples for 16S rRNA amplicon sequencing were collected by individual clean catch at the time of cage changing at 11 and 18 wk of age for both generations. Mice were euthanized when 18 wk old by 100% carbon dioxide inhalation with gradual fill of the chamber, death confirmed by cervical dislocation and intestinal tissue harvested. Intestinal tissue from four female 12 wk of age RF C57BL/6NTac mice from the same barrier as the RF donors, and four male and three female 6 wk of age GF C57BL/6NTac served as reference samples for the gene expression experiments.

### DNA isolation, library building and 16S rRNA amplicon sequencing

DNA isolation of fecal samples, library building and sequencing was performed as previously described^93^. Briefly, samples were randomized and DNA was isolated using PowerLyzer PowerSoil DNA Isolation Kit (MO BIO Laboratories, Carlsbad, CA, USA). The V3 region of the 16S rRNA gene was amplified by PCR as previously described^94^, and amplicons purified using the HighPrep PCR Clean Up System (MAGBIO Genomics Inc., AC-60050). The DNA libraries were multiplexed in batches of 89 in an equimolar ratio and stored at −20 °C until sequencing. The 16S rRNA amplicon libraries were sequenced on the Ion PGM System using a 318-chip, the Ion PGM Template OT2 200 Kit (Thermo Fisher Scientific, A26434) and the Ion PGM Hi-Q Sequencing Kit (Thermo Fisher, A26433). Sequence data are deposited in the Sequence Read Database (SRA) with the accession number SRP158406.

### 16S rRNA amplicon data processing

Handling of 16S rRNA amplicon data was done as previously described^93^. Briefly, the software CLC Genomics Workbench vs. 7.0 (CLC bio, Qiagen, Aarhus, Denmark) was used to demultiplex and trim reads to remove primers, barcodes, low quality sequences (quality score=0.05), ambiguous nucleotides (maximally 2 allowed) and reads below 110 bp and above 180 bp. OTUs were picked de novo using UPARSE algorithm^95^ with a maximum expected error (maxee) rate of 3.5 and no truncation of reads. Chimera filtering was done using the rdp_gold.fa database as ref. ^96^. Taxonomy was assigned in Quantitative Insights Into Microbial Ecology (QIIME^97^) version 1.9.1 to Operational Taxonomic Units (OTUs) with 97% similarity using the Silva 111 reference database^98^. Samples with fewer than 1300 reads were filtered from the data set. In total, 180 fecal samples were included in the study (48 HM B6, 59 MM B6, 67 MM SW, 4 PCR replicates of the HM donor sample, 2 PCR replicates of the MM donor sample) and had a mean of 29449 reads/sample (Min: 1318, Max: 120717, SD: 19735) with 2552 OTUs represented. OTU tables were normalized to 1200 reads/sample (corresponding to ~90% of the sample with fewest reads) for alpha diversity, colonization efficiency and differential abundance analyses. UniFrac^99^ distance matrices with a depth coverage of 1200 reads/sample formed the basis of Principal Coordinates Analyses (PCoA), and 3D PCoA plots were created in EMPeror^100^. For relative abundance, a cut-off threshold for abundance at 0.5% was set.

### qPCR for tissue gene expression

Sections of ileum and colon of approximately 0.5 cm were excised cranially to the ileocecal valve and caudally to the colocecal junction, respectively. Peyer’s patches were excised and discarded from the samples, which were then cleaned from luminal contents, stored in 0.5 ml of RNAlater (Sigma-Aldrich, R0901) and after soaking for 24 hours at +4 °C transferred to storage at −80 °C. Tissue was homogenized in 0.5 ml MagMAX Lysis/Binding Solution Concentrate (Thermo Fisher, AM8500), 3.5 μl β-mercaptoethanol and approximately 0.6 g of glass beads <106 µm (Sigma-Aldrich) using a FastPrep 24 instrument (MP Biomedicals, Santa Ana, CA, United States). Homogenates were stored at −20 °C. Total RNA was extracted using the MagMAX-96 Total RNA Isolation kit (Thermo Fisher, AM1830) according to the manufacturer’s instructions on a MagMAX Express Magnetic Particle Processor (Thermo Fisher). cDNA was synthesized from ∼500 ng total RNA by using the High-Capacity cDNA Reverse Transcriptase kit (Thermo Fisher, 4368814) according to the manufacturer’s instructions. Levels of mRNA were measured by qPCR of *Cd4* (Mm00442754_m1), *Cd8a* (Mm01182107_g1), *Foxp3* (Mm00475162_m1), *Itgax* (Mm00498698_m1), *Cd19* (Mm00515420_m1), *Cd22* (Mm00515432_m1), *Muc1* (Mm00449604_m1), *Muc2* (Mm01276696_m1), *Ocln* (Mm00500912_m1), *Tjp1* (Mm00493699_m1), *Tlr2* (Mm00442346_m19, *Tlr4* (Mm00445273_m1), *Reg3g* (Mm00441127_m1) and *Il17a* (Mm00439619_m1) using TaqMan gene expression assays (Thermo Fisher Scientific) and TaqMan Fast universal PCR Mastermix (Thermo Fisher, 4352042) on cDNA duplicates on a StepOnePlus instrument (Thermo Fisher) as previously described^101^. When both duplicate C_T_ values were returned as undetermined, we artificially set the C_T_ value to 40. If one duplicate was undetermined, and the other was returned with a high C_T_ value (e.g., 35–36), we used the single C_T_ value. Actin beta (*Actb* (Mm00607939_s1)) was used as the endogenous reference gene for normalization of data by defining ΔC_T_ as C_T(target)_ – C_T(reference)_. Relative quantification (RQ) was then calculated as 2^−ΔΔCT^, where ΔΔC_T_ = ΔC_T(sample)_ − ΔC_T(calibrator)_ with the calibrator being the mean ΔC_T_ of samples from GF B6 mice.

### Cytokine and chemokine multiplex

Blood was sampled by cardiac puncture from anaesthetized mice which were subsequently euthanized. The blood was centrifuged at 8000 g for 8 min and plasma was used to measure cytokines with a multiplex V-PLEX mouse cytokine 29-Plex kit (Mesoscale Discovery, Rockville, MD) according to manufacturer’s instructions. IFN-γ, IL-1β, IL-2, IL-4, IL-5, IL-6, IL-9, CXCL1, IL-10, IL-12p70, IL-15, IL17A/F, IL-27p28/IL30, IL-33, CXCL10, CCL2, CCL3, CXCL2, TNF-α, CCL20, IL-22, IL-23, IL-17C, IL-31, IL-21, IL-17F, IL-16, IL-17A, IL-17E/IL-25 were included in the kit. IL-6, IL-17F, IL-21, IL-23, IL-31, IL-9, IL-17A/F, IL-12p70, IL-4 were below detection range. Results were read and analyzed on a MESO QuickPlex SQ120.

### Plasma IgE

IgE levels were measured using Mouse IgE ELISA Kit (Bethyl Laboratories, Montgomery, TX) according to the standard protocol using a 1:20 dilution. Absorbance was measured and analyzed on a spectrophotometer (PowerWave x Microplate Spectrophotometer and KC4 v3.4, Rev 21, Bio-Tek Instruments INC, Winooski, VT).

### Statistical methods

Model assumptions for gene expression and alpha diversity data were assessed by residual plots and Anderson-Darling normality test in Minitab17 Statistical Software (Minitab Ltd., Coventry, UK). Alpha diversity was analyzed by one-way ANOVA with Tukey’s pairwise comparisons in Minitab17. For calculating colonization efficiency, a cut-off of 0.1% abundance was applied and the mean number of different OTUs per group was related to the number of OTUs for the inoculum of that group. Differential abundance analysis was done on tables with OTUs summarized on genus level and filtered from taxa present in less than 25% of the samples by Kruskal-Wallis test with 1000 permutations and Bonferroni correction for multiple comparisons using QIIME’s script group_significance.py. Beta diversity clustering was assessed by ANOSIM (analysis of similarities) with 999 permutations. We considered an R-value of 0.75–1 as strong separation, 0.25–0.75 as moderate separation, and <0.25 as low separation in the ANOSIM tests. Testing UniFrac distance differences to the inoculum was done by the script make_distance_comparison_plots.py using Student’s t-test. For the gene expression experiments, ΔC_T_ values were analyzed in Minitab17 by Student’s t-test. Males and females were analyzed separately, whereas the P and F1 generation were analyzed together, as there were no differences between the generations for any of the targets measured in both generations. GraphPad Prism 6 (GraphPad Software, La Jolla, CA, USA) was used to create RQ and alpha diversity plots. Correlations between gene expression ΔC_T_ values and the absolute, rarefied abundance of OTUs were done by fitting a linear regression model in Minitab17 by using all OTUs as continuous predictors and “Microbiota” and “Sex” as categorical predictors. Subsequently, the model was run with the top-three influencing OTUs on the model to obtain p-values. All analyses were performed on a 95% significance level. Errors are reported as SD.

### Ethics approval and consent to participate

The animal experiments were carried out in accordance with the EU directive 2010/63/EU and the Danish Animal Experimentation Act (LBK 474 from 15/05/2014) and were approved by the Danish Animal Experimentation Inspectorate (Ministry of Environment and Food in Denmark) according to license no. 2012-15-2934-00256. Written informed consent was obtained from the human donor prior to donating the fecal material to the study, as recommended by the Metropolitan Region Science Ethics Committee, which did not request further licensing. Handling and processing of the human fecal sample and other methods employed during the execution of the experiments were carried out in accordance to relevant national guidelines and legislation.

## Supplementary information

Supplementary Dataset 1.

## Data Availability

Sequence data are deposited in the Sequence Read Database (SRA) with the accession number SRP158406.
