# Supplementary material for: Human microbiota-transplanted C57BL/6 mice and offspring display reduced establishment of key bacteria and reduced immune stimulation compared to mouse microbiota-transplantation
Source: Sci Rep. 2020 May 8;10:7805. doi: 10.1038/s41598-020-64703-z (PMC7211022; doi:10.1038/s41598-020-64703-z)
Supplement: Supplementary file 1 — Supplementary Dataset 1. [file 41598_2020_64703_MOESM1_ESM.pdf]

Supplementary Figures and Tables

Human microbiota-transplanted C57BL/6 mice and offspring display reduced establishment of key bacteria and reduced immune stimulation compared to mouse microbiota-transplantation

Randi Lundberg[1,2, 3,*], Martin F. Toft[2,4], Stine B. Metzdorff[1], Camilla H. F. Hansen[1], Tine R. Licht[5], Martin I. Bahl[5], Axel K. Hansen[1]

[1]Department of Veterinary and Animal Sciences, Faculty of Health and Medical Sciences, University of Copenhagen, 1871 Frederiksberg C, Denmark

[2]Internal Research and Development, Taconic Biosciences, 4623 Lille Skensved, Denmark

[3]Current address: Chr. Hansen, 2970 Hoersholm, Denmark

[4]Current address: QM Diagnostics, 6534 AT Nijmegen, The Netherlands

[5]National Food Institute, Technical University of Denmark, 2800 Kgs. Lyngby, Denmark

* Corresponding author: lundberg.randi@gmail.com; https://orcid.org/0000-0002-6646-6036

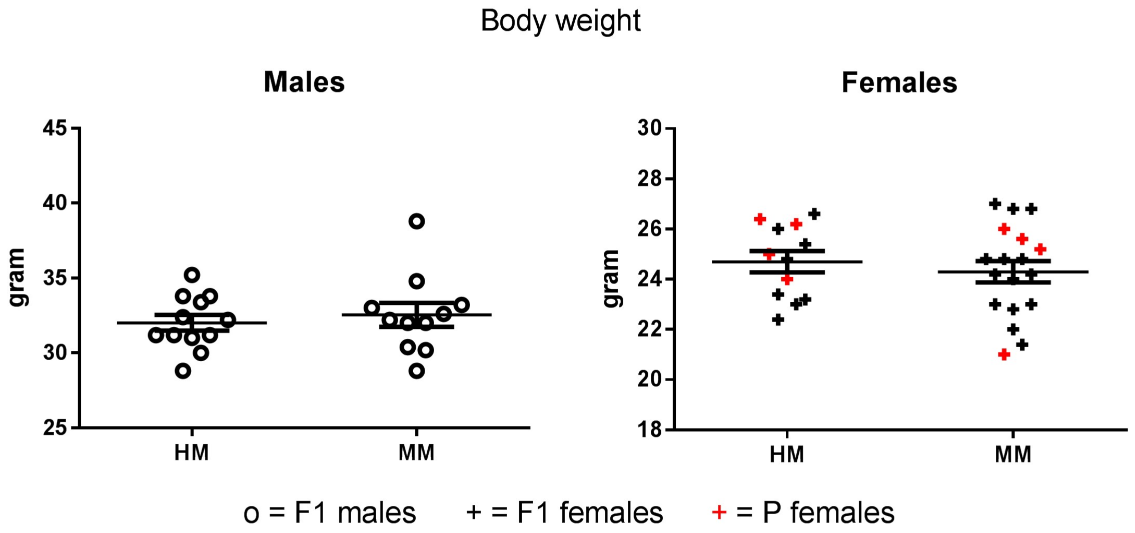

Supplementary Figure S1. Body weight of male and female C57BL/6NTac mice colonized with a human microbta (HM) or mouse microbiota (MM). Students t-test, SEM. P=transplanted parent generation, F1=offspring generation born with microbiota.

OTU #	Hum. inoc.	B6																														
		P 11 wk			P 18 wk			F1 11 wk														F1 18 wk										
1	22%	4%	4%	8%	6%	8%	6%	3%	5%	4%	2%	4%	4%	5%	5%	3%	3%	6%	3%	4%	4%	3%	4%	3%	3%	3%	3%	3%	4%	3%	5%	8%
2	13%	17%	8%	14%	13%	25%	23%	42%	14%	22%	14%	10%	13%	7%	28%	32%	15%	16%	11%	39%	27%	16%	17%	5%	7%	17%	14%	11%	22%	8%	44%	31%
3	11%	<1%	<1%	<1%	<1%	<1%	<1%	0%	0%	0%	0%	0%	0%	0%	0%	0%	0%	<1%	0%	<1%	0%	<1%	<1%	0%	<1%	<1%	0%	0%	<1%	0%	<1%	0%
4	10%	<1%	<1%	1%	<1%	1%	<1%	<1%	<1%	<1%	<1%	<1%	<1%	<1%	<1%	<1%	<1%	<1%	<1%	1%	<1%	2%	<1%	<1%	<1%	<1%	<1%	<1%	<1%	<1%	<1%	1%
5	6%	2%	3%	7%	3%	10%	3%	2%	8%	<1%	<1%	7%	3%	5%	2%	2%	9%	9%	8%	5%	5%	7%	3%	<1%	<1%	1%	<1%	3%	<1%	2%	2%	6%
6	5%	0%	0%	0%	0%	0%	0%	0%	0%	0%	0%	0%	0%	0%	0%	0%	0%	0%	0%	0%	0%	0%	0%	0%	0%	0%	0%	0%	0%	0%	0%	0%
7	5%	0%	<1%	<1%	<1%	<1%	<1%	<1%	<1%	<1%	<1%	<1%	<1%	<1%	<1%	<1%	<1%	<1%	<1%	<1%	<1%	<1%	<1%	<1%	<1%	<1%	<1%	<1%	<1%	<1%	<1%	<1%
8	4%	0%	0%	0%	<1%	0%	0%	0%	0%	<1%	0%	0%	0%	<1%	0%	<1%	0%	0%	0%	0%	0%	0%	0%	0%	0%	0%	0%	0%	0%	0%	0%	0%
9	4%	<1%	<1%	<1%	<1%	<1%	<1%	<1%	<1%	3%	3%	2%	<1%	<1%	<1%	<1%	<1%	2%	3%	1%	<1%	<1%	<1%	<1%	<1%	<1%	<1%	<1%	<1%	<1%	<1%	2%
10	4%	0%	<1%	0%	0%	0%	0%	0%	0%	0%	0%	0%	0%	0%	0%	0%	0%	0%	0%	0%	0%	0%	0%	0%	0%	0%	0%	0%	0%	0%	0%	0%
11	3%	4%	2%	2%	<1%	4%	2%	4%	6%	0%	0%	9%	5%	6%	5%	5%	11%	11%	14%	20%	5%	17%	7%	2%	3%	4%	0%	11%	0%	3%	5%	0%
12	2%	5%	3%	3%	6%	5%	8%	5%	5%	7%	4%	7%	4%	4%	5%	5%	6%	5%	7%	13%	6%	7%	10%	6%	6%	9%	5%	3%	12%	6%	6%	10%
13	2%	0%	0%	0%	0%	0%	0%	0%	0%	0%	0%	0%	0%	0%	0%	0%	0%	0%	0%	0%	0%	0%	0%	0%	0%	0%	0%	0%	0%	0%	0%	0%
14	2%	1%	<1%	1%	<1%	<1%	<1%	0%	0%	<1%	0%	<1%	0%	0%	0%	0%	0%	<1%	0%	0%	0%	<1%	0%	0%	0%	0%	0%	0%	0%	0%	0%	0%
15	1%	1%	3%	4%	3%	2%	3%	1%	4%	4%	4%	3%	5%	5%	3%	6%	3%	4%	3%	3%	4%	7%	2%	3%	3%	3%	4%	2%	2%	4%	3%	2%
16	1%	<1%	<1%	<1%	<1%	<1%	<1%	<1%	<1%	1%	1%	<1%	<1%	<1%	<1%	<1%	1%	<1%	<1%	<1%	<1%	2%	<1%	<1%	<1%	<1%	<1%	<1%	<1%	<1%	<1%	<1%
17	1%	43%	47%	37%	48%	31%	39%	29%	37%	39%	47%	43%	42%	47%	36%	33%	38%	28%	33%	9%	37%	22%	43%	60%	57%	46%	55%	46%	44%	46%	22%	22%
18	<1%	<1%	<1%	<1%	<1%	<1%	<1%	<1%	<1%	<1%	0%	<1%	<1%	<1%	0%	<1%	<1%	<1%	0%	0%	<1%	<1%	<1%	<1%	<1%	<1%	<1%	<1%	<1%	<1%	<1%	0%
19	<1%	<1%	<1%	<1%	<1%	<1%	<1%	<1%	<1%	<1%	<1%	<1%	<1%	<1%	<1%	<1%	<1%	<1%	0%	<1%	<1%	<1%	<1%	<1%	<1%	<1%	<1%	<1%	<1%	<1%	<1%	0%
20	<1%	0%	<1%	0%	0%	0%	<1%	0%	<1%	0%	<1%	0%	0%	<1%	0%	<1%	<1%	0%	<1%	0%	0%	0%	<1%	0%	0%	0%	0%	<1%	0%	0%	0%	0%
21	<1%	<1%	<1%	<1%	<1%	<1%	<1%	<1%	<1%	<1%	<1%	<1%	<1%	<1%	<1%	<1%	<1%	<1%	<1%	<1%	<1%	<1%	<1%	<1%	<1%	<1%	<1%	<1%	<1%	<1%	0%	<1%
22	<1%	2%	1%	1%	<1%	<1%	1%	0%	0%	0%	0%	0%	0%	0%	0%	0%	0%	0%	0%	0%	0%	0%	0%	0%	0%	0%	0%	0%	0%	0%	0%	0%
23	<1%	<1%	<1%	<1%	0%	<1%	0%	<1%	<1%	<1%	<1%	<1%	<1%	<1%	<1%	0%	0%	<1%	1%	<1%	0%	<1%	0%	0%	<1%	0%	<1%	<1%	<1%	<1%	<1%	7%
24	<1%	0%	0%	0%	0%	0%	0%	0%	<1%	0%	0%	0%	0%	<1%	0%	0%	0%	0%	0%	0%	0%	0%	0%	0%	0%	0%	0%	0%	0%	0%	0%	0%
25	<1%	11%	20%	13%	11%	6%	8%	8%	9%	11%	14%	9%	16%	16%	12%	7%	4%	10%	12%	2%	6%	12%	10%	16%	14%	12%	11%	13%	9%	21%	9%	6%
26	<1%	<1%	<1%	<1%	<1%	<1%	<1%	<1%	<1%	<1%	<1%	<1%	<1%	<1%	<1%	<1%	<1%	<1%	<1%	<1%	<1%	<1%	<1%	<1%	<1%	<1%	<1%	0%	<1%	<1%	<1%	<1%
27	<1%	3%	5%	4%	2%	4%	2%	<1%	8%	5%	6%	<1%	4%	1%	2%	2%	7%	3%	<1%	<1%	4%	<1%	2%	1%	<1%	3%	3%	7%	1%	3%	1%	<1%
28	<1%	0%	<1%	0%	0%	0%	0%	0%	<1%	0%	0%	<1%	<1%	0%	0%	0%	0%	0%	0%	0%	0%	0%	0%	0%	0%	0%	0%	0%	0%	0%	0%	0%
29	<1%	0%	0%	0%	0%	0%	0%	0%	0%	0%	0%	0%	0%	0%	0%	0%	0%	0%	0%	0%	0%	0%	0%	0%	0%	0%	0%	0%	0%	0%	0%	0%
30	<1%	0%	0%	0%	0%	0%	0%	<1%	0%	0%	0%	0%	0%	<1%	0%	<1%	0%	0%	0%	0%	0%	0%	0%	0%	<1%	0%	<1%	0%	<1%	0%	0%	0%
31	<1%	3%	2%	3%	5%	2%	3%	3%	1%	3%	3%	3%	2%	2%	<1%	3%	1%	3%	4%	<1%	1%	4%	2%	2%	5%	2%	4%	<1%	4%	3%	2%	1%
32	<1%	<1%	<1%	<1%	<1%	<1%	<1%	<1%	<1%	<1%	<1%	<1%	<1%	<1%	<1%	<1%	<1%	<1%	<1%	0%	<1%	0%	<1%	<1%	<1%	<1%	<1%	0%	<1%	<1%	<1%	0%
33	<1%	<1%	<1%	<1%	<1%	<1%	<1%	<1%	<1%	<1%	<1%	<1%	<1%	<1%	<1%	<1%	<1%	<1%	<1%	<1%	<1%	<1%	<1%	<1%	<1%	<1%	<1%	<1%	<1%	<1%	<1%	<1%
N.d.		<1%	<1%	<1%	<1%	<1%	<1%	<1%	<1%	<1%	<1%	<1%	<1%	<1%	<1%	<1%	<1%	<1%	<1%	<1%	<1%	<1%	<1%	<1%	<1%	<1%	<1%	<1%	<1%	<1%	<1%	<1%
Total	100%	100%																														

Supplementary Table S1. Relative abundance of genera compared to the human microbiota (HM) inoculum in fecal samples from P and F1 B6 mice sampled at 11 and 18 wk of age. B6=C57BL/6NTac, P=transplanted parent generation, F1=offspring generation born with microbiota, Hum.inoc.= human inoculum.

Supplementary Table S1 - continued. List of genera.

1	Firmicutes_Clostridia_Clostridiales_Ruminococcaceae_Other
2	Firmicutes_Clostridia_Clostridiales_Lachnospiraceae_Other
3	Firmicutes_Clostridia_Clostridiales_Lachnospiraceae_Pseudobutyrivibrio
4	Actinobacteria_Coriobacteriia_Coriobacteriales_Coriobacteriaceae_Collinsella
5	Firmicutes_Clostridia_Clostridiales_Lachnospiraceae_Blautia
6	Firmicutes_Clostridia_Clostridiales_Veillonellaceae_Dialister
7	Firmicutes_Clostridia_Clostridiales_Christensenellaceae
8	Firmicutes_Clostridia_Clostridiales_Ruminococcaceae_Faecalibacterium
9	Actinobacteria_Actinobacteria_Bifidobacteriales_Bifidobacteriaceae_Bifidobacterium
10	Firmicutes_Clostridia_Clostridiales_Lachnospiraceae_Shuttleworthia
11	Firmicutes_Clostridia_Clostridiales_Ruminococcaceae_Subdoligranulum
12	Firmicutes_Clostridia_Clostridiales_Other
13	Actinobacteria_Coriobacteriia_Coriobacteriales_Coriobacteriaceae_Slackia
14	Firmicutes_Clostridia_Clostridiales_Lachnospiraceae_Dorea
15	Bacteroidetes_Bacteroidia_Bacteroidales_Rikenellaceae_Alistipes
16	Firmicutes_Erysipelotrichi_Erysipelotrichales_Erysipelotrichaceae_Catenibacterium
17	Bacteroidetes_Bacteroidia_Bacteroidales_Bacteroidaceae_Bacteroides
18	Firmicutes_Clostridia_Clostridiales_Ruminococcaceae_Anaerotruncus
19	Firmicutes_Clostridia_Clostridiales_Ruminococcaceae_Flavonifractor
20	Bacteroidetes_Bacteroidia_Bacteroidales_Prevotellaceae_Prevotella
21	Firmicutes_Bacilli
22	Firmicutes_Clostridia_Clostridiales_Ruminococcaceae_Oscillibacter
23	Firmicutes_Clostridia_Clostridiales_Peptostreptococcaceae
24	Firmicutes_Clostridia_Clostridiales_Lachnospiraceae_Anaerostipes
25	Bacteroidetes_Bacteroidia_Bacteroidales_Other
26	Actinobacteria_Coriobacteriia_Coriobacteriales_Coriobacteriaceae_Other
27	Firmicutes_Other
28	Other
29	Spirochaetes
30	Actinobacteria_Actinobacteria_Other
31	Proteobacteria
32	Cyanobacteria
33	Verrucomicrobia
N.d.=	Not detected in inoculum

OTU #	Mur. inoc.	B6																																					
		P 11 wk				P 18 wk			F1 11 wk																F1 18 wk														
1	52%	23%	17%	23%	30%	37%	22%	26%	19%	41%	12%	13%	10%	22%	29%	32%	19%	13%	61%	30%	6%	9%	14%	28%	31%	55%	41%	30%	49%	47%	30%	30%	39%	43%	60%	47%	63%	44%	64%
2	21%	46%	34%	32%	31%	21%	40%	45%	40%	23%	43%	38%	44%	38%	34%	39%	40%	59%	14%	27%	36%	45%	44%	45%	31%	19%	24%	37%	17%	22%	38%	36%	6%	23%	10%	25%	14%	30%	11%
3	8%	2%	6%	3%	4%	3%	3%	3%	3%	5%	3%	5%	4%	4%	6%	4%	4%	2%	5%	3%	2%	3%	3%	4%	3%	2%	5%	3%	2%	4%	2%	5%	2%	5%	5%	3%	4%	2%	3%
4	6%	7%	18%	13%	12%	14%	20%	11%	12%	8%	17%	18%	10%	7%	8%	11%	13%	4%	4%	5%	4%	3%	15%	5%	10%	9%	8%	10%	6%	8%	11%	12%	2%	9%	11%	4%	7%	4%	8%
5	2%	<1%	<1%	<1%	0%	<1%	0%	2%	<1%	<1%	0%	<1%	<1%	0%	<1%	0%	0%	<1%	0%	0%	0%	0%	0%	<1%	<1%	0%	<1%	<1%	<1%	<1%	<1%	<1%	<1%	<1%	<1%	<1%	<1%	0%	<1%
6	2%	1%	7%	3%	5%	2%	1%	2%	1%	3%	2%	2%	1%	2%	3%	1%	2%	<1%	1%	<1%	<1%	<1%	1%	2%	4%	1%	3%	2%	2%	4%	3%	2%	2%	2%	4%	3%	2%	3%	4%
7	1%	<1%	<1%	<1%	<1%	<1%	<1%	<1%	<1%	<1%	<1%	<1%	<1%	<1%	<1%	<1%	<1%	<1%	<1%	<1%	0%	<1%	<1%	<1%	<1%	<1%	<1%	<1%	<1%	<1%	<1%	<1%	<1%	<1%	<1%	<1%	<1%	<1%	<1%
8	1%	4%	3%	11%	4%	5%	5%	4%	8%	6%	9%	9%	13%	4%	4%	6%	7%	5%	4%	5%	7%	5%	9%	3%	6%	5%	3%	4%	5%	4%	7%	6%	<1%	3%	3%	3%	2%	2%	2%
9	1%	<1%	4%	5%	1%	2%	2%	4%	8%	2%	7%	7%	6%	3%	3%	4%	4%	5%	<1%	2%	4%	2%	5%	2%	1%	3%	<1%	3%	<1%	1%	1%	2%	<1%	1%	<1%	<1%	<1%	<1%	<1%
10	1%	0%	0%	<1%	<1%	0%	0%	0%	<1%	<1%	<1%	<1%	0%	<1%	<1%	0%	0%	<1%	<1%	0%	0%	<1%	0%	0%	<1%	0%	<1%	<1%	<1%	<1%	<1%	<1%	<1%	<1%	<1%	<1%	<1%	<1%	<1%
11	1%	<1%	<1%	2%	<1%	<1%	<1%	<1%	<1%	1%	1%	<1%	<1%	<1%	1%	1%	<1%	<1%	1%	<1%	<1%	<1%	1%	1%	1%	<1%	1%	<1%	<1%	<1%	<1%	<1%	<1%	<1%	<1%	1%	<1%	1%	1%
12	<1%	<1%	<1%	<1%	<1%	<1%	<1%	<1%	<1%	<1%	<1%	<1%	<1%	<1%	<1%	<1%	<1%	<1%	<1%	<1%	0%	<1%	<1%	<1%	<1%	<1%	<1%	<1%	<1%	<1%	<1%	<1%	<1%	<1%	<1%	<1%	<1%	<1%	<1%
13	<1%	<1%	<1%	<1%	<1%	<1%	<1%	<1%	<1%	<1%	<1%	<1%	<1%	<1%	<1%	<1%	<1%	<1%	<1%	<1%	<1%	<1%	<1%	<1%	<1%	<1%	<1%	<1%	3%	<1%	<1%	<1%	<1%	<1%	<1%	<1%	<1%	<1%	<1%
14	<1%	1%	1%	<1%	<1%	<1%	1%	<1%	<1%	<1%	<1%	<1%	<1%	<1%	2%	<1%	<1%	<1%	2%	<1%	<1%	<1%	<1%	<1%	<1%	<1%	<1%	<1%	<1%	<1%	<1%	<1%	2%	<1%	<1%	<1%	<1%	1%	2%
15	<1%	<1%	<1%	<1%	<1%	<1%	<1%	<1%	<1%	<1%	<1%	<1%	<1%	<1%	<1%	<1%	<1%	<1%	<1%	<1%	<1%	<1%	<1%	<1%	1%	<1%	<1%	<1%	1%	<1%	<1%	<1%	<1%	<1%	<1%	<1%	<1%	<1%	<1%
16	<1%	13%	7%	7%	10%	13%	4%	1%	6%	6%	4%	7%	9%	17%	8%	<1%	10%	11%	6%	27%	38%	30%	6%	6%	9%	3%	11%	7%	14%	6%	3%	4%	43%	8%	2%	11%	4%	10%	2%
17	<1%	0%	0%	0%	0%	0%	0%	0%	0%	0%	0%	0%	0%	0%	0%	0%	0%	0%	0%	0%	0%	0%	0%	0%	<1%	0%	0%	<1%	0%	0%	0%	0%	<1%	0%	0%	<1%	0%	0%	0%
18	<1%	1%	1%	1%	<1%	<1%	<1%	<1%	<1%	<1%	<1%	<1%	<1%	<1%	<1%	<1%	<1%	<1%	<1%	<1%	<1%	<1%	<1%	<1%	<1%	<1%	<1%	<1%	<1%	<1%	<1%	<1%	<1%	<1%	<1%	<1%	<1%	<1%	<1%
19	<1%	0%	0%	0%	0%	0%	0%	0%	0%	0%	0%	0%	0%	0%	0%	0%	0%	0%	0%	0%	0%	0%	0%	0%	0%	0%	0%	0%	0%	0%	0%	0%	0%	0%	0%	0%	0%	0%	0%
20	<1%	<1%	<1%	<1%	<1%	0%	<1%	<1%	<1%	<1%	<1%	<1%	0%	<1%	<1%	<1%	0%	<1%	0%	0%	0%	0%	0%	<1%	0%	0%	<1%	0%	0%	<1%	0%	<1%	0%	0%	<1%	0%	<1%	<1%	0%
21	<1%	<1%	<1%	<1%	<1%	<1%	<1%	<1%	<1%	<1%	<1%	<1%	<1%	<1%	<1%	<1%	<1%	<1%	<1%	<1%	<1%	<1%	<1%	<1%	<1%	<1%	<1%	<1%	<1%	<1%	<1%	<1%	<1%	<1%	0%	<1%	<1%	<1%	<1%
22	<1%	0%	<1%	0%	<1%	<1%	<1%	0%	<1%	<1%	<1%	<1%	<1%	<1%	<1%	<1%	<1%	<1%	<1%	0%	<1%	<1%	<1%	<1%	<1%	<1%	<1%	<1%	<1%	<1%	1%	<1%	<1%	<1%	<1%	<1%	<1%	<1%	<1%
23	<1%	0%	0%	0%	0%	0%	0%	0%	0%	0%	0%	0%	0%	0%	0%	0%	0%	0%	0%	0%	0%	0%	0%	0%	0%	0%	0%	0%	0%	0%	0%	0%	0%	0%	0%	0%	0%	0%	0%
24	<1%	0%	0%	0%	0%	0%	0%	0%	0%	0%	0%	0%	0%	0%	0%	0%	0%	0%	0%	0%	0%	0%	0%	0%	0%	0%	0%	0%	0%	0%	0%	0%	0%	0%	0%	0%	0%	0%	0%
25	<1%	0%	0%	<1%	<1%	<1%	<1%	<1%	<1%	<1%	<1%	<1%	<1%	<1%	<1%	<1%	<1%	0%	<1%	<1%	0%	0%	0%	<1%	0%	<1%	<1%	<1%	<1%	<1%	<1%	<1%	<1%	<1%	0%	<1%	<1%	<1%	0%
N.d.		<1%	<1%	<1%	<1%	0%	<1%	0%	<1%	<1%	0%	<1%	<1%	<1%	<1%	<1%	<1%	<1%	<1%	0%	<1%	<1%	0%	0%	<1%	0%	<1%	<1%	<1%	<1%	<1%	<1%	<1%	<1%	<1%	<1%	<1%	<1%	<1%
Total	100%	100%																																					

Supplementary Table S2. Relative abundance of genera compared to the mouse microbiota (MM) inoculum in fecal samples from P and F1 B6 mice sampled at 11 and 18 wk of age. B6=C57BL/6NTac, P=transplanted parent generation, F1=offspring generation born with microbiota. Mur.inoc.=murine inoculum.

Supplementary Table S2 - continued. List of genera.

1	Firmicutes_Clostridia_Clostridiales_Lachnospiraceae_Other
2	Bacteroidetes_Bacteroidia_Bacteroidales_S24-7
3	Firmicutes_Clostridia_Clostridiales_Ruminococcaceae_Other
4	Bacteroidetes_Bacteroidia_Bacteroidales_Rikenellaceae_Alistipes
5	Firmicutes_Clostridia_Clostridiales_Lachnospiraceae_Moryella
6	Firmicutes_Clostridia_Clostridiales_Other
7	Firmicutes_Clostridia_Clostridiales_Ruminococcaceae_Anaerotruncus
8	Bacteroidetes_Bacteroidia_Bacteroidales_Other
9	Bacteroidetes_Bacteroidia_Bacteroidales_Prevotellaceae
10	Firmicutes_Clostridia_Clostridiales_Lachnospiraceae_Shuttleworthia
11	Firmicutes_Clostridia_Clostridiales_Family_XIII_Incertae_Sedis
12	Firmicutes_Clostridia_Clostridiales_Ruminococcaceae_Flavonifractor
13	Firmicutes_Clostridia_Clostridiales_Lachnospiraceae_Roseburia
14	Actinobacteria_Coriobacteriia_Coriobacteriales_Coriobacteriaceae
15	Proteobacteria_Deltaproteobacteria_Desulfovibrionales_Desulfovibrionaceae_Desulfovibrio
16	Firmicutes_Bacilli_Lactobacillales_Lactobacillaceae_Lactobacillus
17	Firmicutes_Clostridia_Clostridiales_Lachnospiraceae_Johnsonella
18	Firmicutes_Other
19	Deferribacteres
20	Proteobacteria_Other
21	Actinobacteria_Other
22	Tenericutes
23	Cyanobacteria
24	Verrucomicrobia
25	Other
N.d.=	Not detected in inoculum

OTU #	Mur. inoc.	SW																												
		P 11 wk	P 18 wk			F1 11 wk																								
1	52%	15%	40%	9%	55%	50%	10%	69%	41%	23%	66%	29%	3%	52%	51%	43%	37%	64%	15%	14%	8%	30%	22%	10%	15%	54%	56%	54%	59%	6%
2	21%	52%	21%	72%	5%	5%	12%	3%	15%	46%	4%	38%	60%	3%	16%	9%	22%	7%	52%	18%	44%	16%	20%	48%	24%	<1%	17%	19%	10%	60%
3	8%	2%	6%	1%	5%	7%	3%	6%	5%	3%	4%	5%	3%	3%	6%	4%	9%	3%	3%	3%	2%	7%	9%	1%	4%	6%	4%	5%	8%	1%
4	6%	12%	14%	6%	14%	13%	12%	4%	14%	11%	9%	7%	11%	8%	10%	12%	10%	14%	6%	13%	14%	17%	14%	10%	20%	5%	8%	11%	10%	3%
5	2%	0%	<1%	<1%	<1%	<1%	0%	<1%	0%	0%	5%	<1%	0%	4%	<1%	<1%	0%	0%	<1%	<1%	0%	<1%	<1%	0%	0%	<1%	<1%	0%	0%	<1%
6	2%	2%	5%	<1%	7%	6%	1%	5%	3%	1%	2%	4%	3%	3%	3%	3%	5%	4%	1%	1%	<1%	5%	1%	1%	1%	5%	3%	4%	3%	<1%
7	1%	<1%	<1%	<1%	<1%	<1%	<1%	<1%	<1%	<1%	<1%	<1%	<1%	<1%	<1%	<1%	<1%	<1%	<1%	<1%	<1%	<1%	<1%	<1%	<1%	<1%	<1%	<1%	<1%	<1%
8	1%	7%	4%	2%	1%	<1%	13%	1%	3%	5%	2%	5%	17%	3%	3%	5%	3%	3%	3%	3%	7%	8%	3%	4%	16%	<1%	2%	2%	3%	2%
9	1%	<1%	<1%	<1%	<1%	<1%	<1%	<1%	<1%	<1%	<1%	<1%	2%	<1%	<1%	<1%	1%	<1%	1%	<1%	1%	2%	<1%	<1%	<1%	<1%	<1%	<1%	<1%	<1%
10	1%	0%	<1%	<1%	<1%	0%	0%	0%	0%	0%	<1%	<1%	0%	0%	<1%	<1%	<1%	<1%	0%	0%	0%	<1%	<1%	0%	0%	<1%	0%	0%	<1%	0%
11	1%	<1%	<1%	<1%	<1%	<1%	1%	1%	<1%	<1%	<1%	1%	<1%	<1%	<1%	<1%	1%	<1%	<1%	<1%	<1%	<1%	<1%	<1%	<1%	<1%	<1%	<1%	<1%	<1%
12	<1%	<1%	<1%	<1%	<1%	<1%	<1%	<1%	<1%	<1%	<1%	<1%	<1%	<1%	<1%	<1%	<1%	<1%	<1%	<1%	<1%	<1%	<1%	<1%	<1%	<1%	<1%	<1%	<1%	<1%
13	<1%	<1%	<1%	<1%	<1%	<1%	<1%	<1%	<1%	<1%	<1%	<1%	<1%	<1%	<1%	<1%	<1%	<1%	<1%	<1%	<1%	<1%	<1%	0%	<1%	<1%	<1%	<1%	<1%	<1%
14	<1%	1%	<1%	<1%	<1%	<1%	<1%	<1%	1%	<1%	<1%	1%	<1%	1%	1%	1%	<1%	<1%	<1%	1%	<1%	<1%	2%	1%	<1%	<1%	<1%	<1%	<1%	3%
15	<1%	<1%	<1%	<1%	<1%	<1%	<1%	2%	<1%	<1%	1%	<1%	<1%	1%	2%	2%	<1%	<1%	<1%	<1%	<1%	<1%	<1%	<1%	<1%	<1%	2%	2%	<1%	<1%
16	<1%	6%	6%	6%	9%	14%	45%	6%	16%	7%	3%	5%	<1%	19%	5%	19%	8%	2%	12%	45%	22%	11%	26%	23%	17%	24%	4%	2%	2%	11%
17	<1%	0%	0%	0%	0%	0%	0%	0%	0%	0%	0%	0%	0%	0%	0%	<1%	0%	0%	0%	0%	0%	0%	<1%	0%	0%	0%	0%	0%	0%	0%
18	<1%	<1%	<1%	<1%	<1%	<1%	<1%	<1%	<1%	<1%	<1%	1%	<1%	<1%	<1%	<1%	1%	<1%	<1%	<1%	<1%	<1%	<1%	<1%	<1%	<1%	<1%	<1%	<1%	3%
19	<1%	0%	0%	0%	0%	0%	0%	0%	0%	0%	0%	0%	0%	0%	0%	0%	0%	0%	0%	0%	0%	0%	0%	0%	0%	0%	0%	0%	0%	0%
20	<1%	<1%	<1%	<1%	0%	0%	<1%	<1%	<1%	<1%	<1%	<1%	<1%	<1%	<1%	0%	<1%	0%	<1%	<1%	<1%	<1%	<1%	<1%	<1%	0%	<1%	0%	<1%	<1%
21	<1%	<1%	<1%	<1%	<1%	<1%	<1%	<1%	0%	<1%	<1%	<1%	0%	<1%	<1%	<1%	<1%	<1%	<1%	<1%	<1%	<1%	<1%	<1%	<1%	0%	<1%	<1%	0%	<1%
22	<1%	0%	<1%	<1%	<1%	<1%	<1%	<1%	<1%	<1%	<1%	<1%	<1%	<1%	<1%	<1%	<1%	0%	<1%	<1%	<1%	<1%	<1%	0%	<1%	<1%	<1%	0%	<1%	<1%
23	<1%	0%	0%	0%	0%	0%	0%	0%	0%	0%	0%	0%	0%	0%	0%	0%	0%	0%	0%	0%	0%	0%	0%	0%	0%	0%	0%	0%	0%	0%
24	<1%	0%	0%	0%	0%	0%	0%	0%	0%	0%	0%	0%	<1%	0%	0%	0%	0%	0%	0%	0%	0%	0%	0%	0%	0%	0%	0%	0%	0%	0%
25	<1%	0%	<1%	<1%	<1%	<1%	<1%	<1%	<1%	<1%	0%	<1%	0%	<1%	<1%	<1%	<1%	<1%	<1%	<1%	<1%	<1%	<1%	<1%	<1%	<1%	<1%	<1%	<1%	<1%
N.d.		<1%	<1%	<1%	<1%	<1%	<1%	<1%	<1%	<1%	<1%	<1%	<1%	<1%	<1%	<1%	<1%	<1%	5%	<1%	<1%	<1%	<1%	<1%	<1%	<1%	<1%	<1%	<1%	8%
Total	100%	100%																												

OTU #	Mur. inoc.	SW																
		F1 18 wk																
1	52%	27%	40%	47%	12%	57%	31%	34%	51%	45%	38%	24%	58%	34%	18%	24%	44%	25%
2	21%	16%	10%	11%	45%	4%	11%	36%	7%	9%	13%	10%	6%	15%	29%	29%	14%	29%
3	8%	8%	3%	6%	3%	6%	6%	6%	7%	7%	5%	2%	7%	6%	5%	3%	5%	3%
4	6%	30%	3%	14%	16%	14%	27%	8%	11%	20%	11%	6%	11%	26%	29%	5%	17%	17%
5	2%	0%	<1%	<1%	<1%	2%	<1%	<1%	<1%	<1%	<1%	0%	1%	<1%	<1%	<1%	<1%	0%
6	2%	6%	2%	6%	1%	7%	2%	4%	6%	5%	3%	<1%	6%	4%	2%	2%	4%	2%
7	1%	<1%	<1%	<1%	<1%	<1%	<1%	<1%	<1%	<1%	<1%	<1%	<1%	<1%	<1%	<1%	<1%	<1%
8	1%	7%	1%	3%	6%	3%	7%	3%	2%	3%	4%	2%	2%	5%	6%	<1%	3%	2%
9	1%	<1%	<1%	<1%	1%	<1%	<1%	2%	<1%	<1%	<1%	<1%	<1%	<1%	1%	<1%	<1%	<1%
10	1%	0%	<1%	0%	0%	0%	0%	0%	0%	0%	<1%	0%	<1%	0%	<1%	<1%	<1%	0%
11	1%	<1%	<1%	<1%	<1%	1%	<1%	<1%	<1%	<1%	1%	<1%	<1%	<1%	<1%	<1%	<1%	<1%
12	<1%	<1%	<1%	<1%	<1%	<1%	<1%	<1%	<1%	<1%	<1%	<1%	<1%	<1%	<1%	<1%	<1%	<1%
13	<1%	<1%	<1%	<1%	<1%	<1%	<1%	<1%	<1%	<1%	<1%	<1%	<1%	<1%	<1%	<1%	<1%	<1%
14	<1%	<1%	2%	<1%	<1%	<1%	<1%	<1%	1%	<1%	2%	2%	<1%	<1%	1%	3%	<1%	<1%
15	<1%	3%	<1%	1%	<1%	1%	<1%	<1%	2%	1%	<1%	<1%	1%	<1%	<1%	<1%	2%	<1%
16	<1%	<1%	34%	8%	13%	4%	11%	4%	8%	5%	21%	51%	5%	7%	6%	28%	7%	17%
17	<1%	0%	0%	0%	0%	0%	0%	0%	0%	0%	0%	0%	<1%	0%	0%	<1%	0%	0%
18	<1%	<1%	<1%	<1%	<1%	<1%	<1%	<1%	2%	1%	<1%	<1%	<1%	<1%	<1%	2%	<1%	1%
19	<1%	0%	0%	0%	0%	0%	0%	0%	0%	0%	0%	0%	0%	0%	0%	0%	0%	0%
20	<1%	<1%	0%	0%	<1%	<1%	<1%	<1%	<1%	<1%	<1%	<1%	<1%	<1%	<1%	<1%	0%	0%
21	<1%	<1%	<1%	<1%	<1%	<1%	<1%	<1%	<1%	<1%	<1%	<1%	<1%	<1%	<1%	<1%	<1%	<1%
22	<1%	<1%	<1%	<1%	<1%	<1%	<1%	<1%	<1%	<1%	<1%	<1%	<1%	<1%	<1%	<1%	<1%	<1%
23	<1%	0%	0%	0%	0%	<1%	0%	0%	0%	0%	0%	0%	0%	0%	0%	0%	0%	0%
24	<1%	0%	0%	0%	0%	0%	0%	0%	0%	0%	0%	0%	0%	0%	0%	0%	0%	0%
25	<1%	<1%	<1%	<1%	<1%	<1%	<1%	<1%	<1%	<1%	<1%	<1%	<1%	<1%	<1%	<1%	<1%	<1%
N.d.		<1%	<1%	<1%	<1%	<1%	<1%	<1%	<1%	<1%	<1%	<1%	<1%	<1%	<1%	2%	<1%	<1%
Total	100%	100%																

Supplementary Table S3. Relative abundance of genera compared to the mouse microbiota (MM) inoculum in fecal samples from P and F1 SW mice sampled when 11 and 18 wk old. SW=Tac:SW (Swiss Webster), P=transplanted parent generation, F1=offspring generation born with microbiota. Mur.inoc.=murine inoculum.

Supplementary Table S3 - continued. List of genera.

1	Firmicutes_Clostridia_Clostridiales_Lachnospiraceae_Other
2	Bacteroidetes_Bacteroidia_Bacteroidales_S24-7
3	Firmicutes_Clostridia_Clostridiales_Ruminococcaceae_Other
4	Bacteroidetes_Bacteroidia_Bacteroidales_Rikenellaceae_Alistipes
5	Firmicutes_Clostridia_Clostridiales_Lachnospiraceae_Moryella
6	Firmicutes_Clostridia_Clostridiales_Other
7	Firmicutes_Clostridia_Clostridiales_Ruminococcaceae_Anaerotruncus
8	Bacteroidetes_Bacteroidia_Bacteroidales_Other
9	Bacteroidetes_Bacteroidia_Bacteroidales_Prevotellaceae
10	Firmicutes_Clostridia_Clostridiales_Lachnospiraceae_Shuttleworthia
11	Firmicutes_Clostridia_Clostridiales_Family_XIII_Incertae_Sedis
12	Firmicutes_Clostridia_Clostridiales_Ruminococcaceae_Flavonifractor
13	Firmicutes_Clostridia_Clostridiales_Lachnospiraceae_Roseburia
14	Actinobacteria_Coriobacteriia_Coriobacteriales_Coriobacteriaceae
15	Proteobacteria_Deltaproteobacteria_Desulfovibrionales_Desulfovibrionaceae_Desulfovibrio
16	Firmicutes_Bacilli_Lactobacillales_Lactobacillaceae_Lactobacillus
17	Firmicutes_Clostridia_Clostridiales_Lachnospiraceae_Johnsonella
18	Firmicutes_Other
19	Deferribacteres
20	Proteobacteria_Other
21	Actinobacteria_Other
22	Tenericutes
23	Cyanobacteria
24	Verrucomicrobia
25	Other
N.d.=	Not detected in inoculum
